# The Contribution of Fluoride to the Pathogenesis of Eye Diseases: Molecular Mechanisms and Implications for Public Health

**DOI:** 10.3390/ijerph16050856

**Published:** 2019-03-08

**Authors:** Declan Timothy Waugh

**Affiliations:** EnviroManagement Services, 11 Riverview, Doherty’s Rd, Bandon, P72 YF10 Co. Cork, Ireland; declan@enviro.ie; Tel.: +353-23-884-1933

**Keywords:** fluoride, age-related macular degeneration, cataract, glaucoma, molecular mechanisms, heat shock proteins, FoxO proteins, BCL-2, Na+, K+-ATPase, NF-kB, Nrf2, IL-6, diabetes, down syndrome, schizophrenia

## Abstract

This study provides diverse lines of evidence demonstrating that fluoride (F) exposure contributes to degenerative eye diseases by stimulating or inhibiting biological pathways associated with the pathogenesis of cataract, age-related macular degeneration and glaucoma. As elucidated in this study, F exerts this effect by inhibiting enolase, τ-crystallin, Hsp40, Na^+^, K^+^-ATPase, Nrf2, γ -GCS, HO-1 Bcl-2, FoxO1, SOD, PON-1 and glutathione activity, and upregulating NF-κB, IL-6, AGEs, HsP27 and Hsp70 expression. Moreover, F exposure leads to enhanced oxidative stress and impaired antioxidant activity. Based on the evidence presented in this study, it can be concluded that F exposure may be added to the list of identifiable risk factors associated with pathogenesis of degenerative eye diseases. The broader impact of these findings suggests that reducing F intake may lead to an overall reduction in the modifiable risk factors associated with degenerative eye diseases. Further studies are required to examine this association and determine differences in prevalence rates amongst fluoridated and non-fluoridated communities, taking into consideration other dietary sources of F such as tea. Finally, the findings of this study elucidate molecular pathways associated with F exposure that may suggest a possible association between F exposure and other inflammatory diseases. Further studies are also warranted to examine these associations.

## 1. Introduction

Age-related macular degeneration (AMD), cataracts and glaucoma are the leading causes of eye diseases and blindness worldwide. AMD is caused by progressive degeneration of retinal pigment epithelial (RPE) cells and neural retina. AMD is the leading cause for irreversible damage of the vision of people over the age of fifty [1]. The pathogenesis of AMD, which covers a complex interaction of genetic and environmental factors, is strongly associated with chronic oxidative stress that ultimately leads to protein damage and degeneration of RPE [2]. Among the risk factors for AMD are diet, smoking, obesity, hypertension, cardiovascular disease and diabetes [3,4,5,6,7,8,9,10]. Cataracts result from the deposition of aggregated proteins in the eye lens and lens fibre cells plasma membrane damage which causes clouding of the lens, light scattering, and obstruction of vision [11]. Cataract is a multifactorial disease associated with age, diet, smoking, environmental exposure to UVB radiation and inflammatory degenerative diseases such as diabetes, asthma or chronic bronchitis and cardiovascular disease [12,13,14,15]. A recent meta-analysis also found that hypertension increases the risk of cataract [16]. It is important to note that a significantly higher prevalence of cataract is found in individuals with Down syndrome [17,18,19,20], schizophrenia [21] and diabetes [22]. Worldwide, cataract remains the predominant cause of blindness and moderate to severe visual impairment (MSVI) and was the second most common cause of blindness in 2010, after macular degeneration, in five world regions (high income Asia Pacific, Australasia, Western Europe, Southern Latin America, and high-income North America). Overall, one in three blind people was blind due to cataract, and one of six visually impaired people was visually impaired due to cataract in 2010 [23]. Glaucoma can be viewed as neurodegenerative disease involving a progressive loss of retinal ganglion cells (RGC) and characteristic changes in neuroretinal rim tissue in the optic nerve head (ONH) which are accompanied by visual field loss [24]. Hypertension and diabetes are associated with increased risk of glaucoma [25].

From a population health perspective, degenerative eye diseases place a significant burden on society and the public health system. In the Republic of Ireland (RoI), it has been estimated that there were nearly 224,832 people with vision impairment and blindness in 2010. The most common causes of blindness were macular degeneration, glaucoma and cataracts. The total economic cost of vision impairment and blindness was estimated to be €2.14 billion in 2010, which is projected to rise to nearly €2.67 billion by 2020 [26]. In 2016, some 218,000 cataract surgeries took place in the RoI [27], however, due to delays performing surgery and patient waiting lists an increasing number of Irish citizens are travelling abroad for cataract operations A recent study found that the prevalence of AMD in adults over 50 years of age in the RoI was 7.2% [28]. Elsewhere, Nolan et al. reported that the prevalence of early AMD was 28% in a randomly selected sample of Irish subjects over 50 years of age [29]. 

In the EUREYE Study the prevalence of AMD in persons 65 years and older in seven European countries including, Bergen, Norway; Tallinn, Estonia; Belfast, Northern Ireland, U.K.; Paris-Creteil, France; Verona, Italy; Thessaloniki, Greece; and Alicante, Spain was 3.3%, with no significant differences found among the participating countries. The prevalence of AMD in Belfast, Northern Ireland among person over 65 years was 3.77% [30]. More recently, Colijn et al. reported in 2017 that the prevalence of early AMD among participants from 10 countries in Europe including Estonia, France, Germany, Greece, Italy, Northern Ireland, Norway, Netherlands, Spain, Portugal, and the U.K. was 3.5% among persons aged 55–59 years [31]. Previously, Owen et al. reported that the prevalence of AMD in the U.K. among people aged 50 years or over is 2.4% (from a meta-analysis applied to UK 2007–2009 population data). This increases to 4.8% in people aged 65 years or over, and 12.2% in people aged 80 years or over [32]. In Iceland, it has been reported that the prevalence of AMD among subjects 50 years and older is 2.3% [33], which is similar to that reported in Norway among subjects 51 years and older (2.9%) [34]. In the Netherlands, Klein et al. reported a prevalence of 1.2% for AMD among the population under 85 years of age [35]. In the Japanese population, the prevalence of early AMD in the Funagata Study was 3.5% among all participants 35 years and older and 4.3% in those 50 years and over [36].

Similar to the RoI, significantly higher prevalence rates of AMD have been reported in the United States (U.S.). For example, Klein et al. reported that the prevalence of AMD among persons over 40 years was 6.5%. Among non-Hispanic whites the prevalence was 7.3% [37]. Previous US studies reported that the prevalence of early AMD among non-Hispanic whites was 14.7% among adults aged 60 years and over [38]. In addition to AMD, the prevalence of cataracts among individuals over 40 years of age in the US was 17.2% in 2004 [39]. Furthermore, by 2020, over 30.1 million people are projected to have cataracts in the U.S. [39]. In 2015, some 9000 ophthalmic surgeons were performing 3.6 million cataract surgeries in the U.S. [40]. The average cost of cataract surgery in the U.S. has been reported to be US $2525 [41]. This suggests that the costs associated with cataract surgery alone in the USA may be in excess of 9 billion dollars annually. Elsewhere it has recently been reported that the economic cost of treating diabetes is over 176 billion dollars a year in the United States, of which over 20% is spent on the ophthalmic complications [42]. As previously noted, diabetes is associated with significantly increased risk of cataract, AMD and glaucoma. 

A higher prevalence rate of AMD has also been reported in Australia. Recently Keel et al. reported that the weighted prevalence among nonindigenous Australians 50 years and older was 14.8% for early AMD and 10.5% for intermediate AMD. Among indigenous Australians 40 years and older, the weighted prevalence was 13.8% for early AMD and 5.7% for intermediate AMD. Among persons aged 70–79 years the prevalence was 17.4% for early AMD and 14.7% for intermediate AMD [43]. In Australia a 2.6-fold increase in the total number of cataract procedures was also documented between 1985 to 1994 [44]. Moreover, the rate of cataract surgery per thousand persons aged 65 years or older doubled between the mid-1980s and mid-2000s [45]. McCarthy et al. previously reported that the prevalence of cataracts among Australians over 40 years of age was 12.6% [46]. Rochtchina et al. reported that by the year 2021 the number of people affected by cataract in Australia will increase by 63%, due to population aging [47]. In New Zealand, the prevalence of AMD is uncertain due to a lack of appropriate studies, but it was estimated in 2014 that it affected 10% of people aged 45–85 years, and 38% of people aged over 85 years [48]. It was further estimated that AMD accounts for 48% of cases of blindness among adults aged 50 years and older in New Zealand and causes approximately 400–500 new cases of blindness per year [49,50]. Moreover, it is estimated that 370,000 of the population have cataracts and 30,000 cataract surgeries are performed every year in New Zealand, [51]. 

As elucidated above, evidence tentatively suggests that the overall prevalence of degenerative eye diseases, particularly AMD, is significantly higher in developed countries with water fluoridation; including, the RoI, U.S., Australia and New Zealand, than in other developed countries without fluoridation of drinking water. Within Europe, the 3-fold differences in prevalence rates for AMD between the RoI the U.K. and mainland Europe are intriguing, especially considering the proximity of the RoI to the U.K. and the shared landmass of the island of Ireland, along with similarities in diet and genetic makeup. It is important to highlight that drinking water is artificially fluoridated in the RoI since 1964, with currently over 80% of households provided with fluoridated water compared to <10% in the U.K. In mainland Europe, drinking water is fluoridated in only one small region, principally the Basque country of Spain since 1988. 

Evidence in support of the hypothesis that fluoride (F) intake may be a contributory factor to degenerative eye diseases include several studies documenting that F can accumulate to high concentrations in the eye contributing to retinal toxicity [52,53,54,55,56,57]. An association between chronic F exposure and cataracts has also been reported in human [58,59,60,61,62,63] and animal studies [64,65]. Furthermore, early in vitro studies by Nordmann et al. using calf lens confirmed that a blockage of the breakdown of sugars by F is followed by cataracts [66]. Further in vitro studies examining metabolism of the lens and of retina identified that F is an enzyme inhibitor in ocular tissue [67,68,69]. Consistent with this finding, early research by Dickens and Simer observed that F significantly inhibited glycolysis in the retina [70]. Previous human studies have also reported an association between chronic F intake and iridocorneal angle hyperpigmentation and open angle glaucoma [71]. However, there is a paucity of qualitative research in epidemiology in western countries to examine the possible association between F intake, water fluoridation and degenerative eye diseases and no study until now has elucidated the molecular mechanisms by which F intake may increase the likelihood of AMD, cataracts or glaucoma. Given the high societal and economic costs of eye diseases in developed countries and globally, a review of modifiable risk factors and the molecular mechanisms by which chronic F exposure may contribute to degenerative eye diseases is therefore warranted. Although much information has become available in recent decades, evidence of a causal relationship requires plausible biological mechanisms by which chronic F exposure may contribute to degenerative eye diseases. Consequently, the purpose of the present study is therefore to elucidate for the first time the key biological mechanisms underlying how F exposure may contribute to degenerative eye diseases including AMD, cataracts and glaucoma. This study therefore provides important insights into the molecular mechanisms by which F intake contributes to degenerative eye diseases and complements the findings of previous human and animal studies making it possible to reach definite conclusions. An understanding of the mechanisms can also elucidate the conditions under which dietary intervention will be most effective and help to identify target populations who may receive optimal benefits.

## 2. The Role of Fluoride in Oral Health and Dietary Sources of Fluoride

Today, community water fluoridation and F toothpaste are considered the most common sources of F exposure in the U.S. [72]. In countries such as the RoI, U.K., Australia and New Zealand, where habitual tea drinking is commonplace, the major dietary source of F is tea consumption [73,74,75]. In addition to tea, fluoridated water, and toothpaste other sources of F exposure include other beverages produced from fluoridated water (beers, coffee, soft drinks, and fruit juices); pesticide residues in foods, foods processed or cooked in fluoridated water; foods grown in soil containing F or irrigated with fluoridated water; consumption of foods with elevated F levels (i.e., seafood and processed chicken); foods cooked in Teflon cookware; tobacco consumption; use of fluoridated mouthwash; use of medical inhalers containing fluoridated gases, and fluoridated medications, in addition to other environmental or occupational exposures to F [75]. 

F has no known essential function in human growth and development and no signs of F deficiency have been identified [76]. However, F is considered to have played a major role in the reduction of dental caries in the past decades in the industrialized countries. It is added as an anti-caries agent to a variety of vehicles, particularly drinking water and toothpastes. Though F is not essential nutrient, current views of its anti-caries action suggest that it is beneficial in the prevention of dental caries when applied topically on the tooth surface and ingestion is not required [77,78]. However, caries is not a F deficiency disease [76]. 

## 3. Molecular and Biochemical Markers Relevant to the Pathophysiology of Eye Diseases

Knowledge of type-specific risk factors of degenerative eye diseases is important for the investigation of association between chronic F exposure and eye diseases.

### 3.1. The Role of Oxidative Stress and Antioxidants in Eye Disease

Overproduction of reactive oxygen species (ROS) or dysfunction of anti-oxidative enzymes can result in oxidative stress and lead to cellular damages [79,80]. When anti-oxidant defence mechanisms are impaired the mechanisms by which increased free radical production and oxidative stress can cause cellular injury increase [81]. Excessive oxidative damage due to ROS and oxidative stress is a major factor in the pathogenesis of many vision-impairing diseases such as age-related macular degeneration, glaucomatous neurodegeneration, cataracts and diabetic retinopathy [82,83,84,85,86,87,88,89,90,91,92,93,94,95].

The lens is able to defend itself against oxidation using antioxidants from either enzymatic or nonenzymatic systems to maintain lens transparency [82,96]. ROS are degraded through the enzymatic activities of superoxide dismutase (SOD), catalase (CAT), and glutathione (GSH) and peroxidases [97,98,99,100,101]. Elevated oxidative stress and a decrease in antioxidant capacity results in retinal dysfunction and cell loss leading to visual impairment [102]. It has also been shown that the antioxidant enzymes such as superoxide dismutase (SOD) in cataractous lenses are decreased, suggesting a role of antioxidant enzymes in the genesis of cataracts [103,104,105,106]. In a study conducted in Turkey, it was reported that serum SOD concentrations were significantly lower and lipid peroxidation products significantly higher in patients with AMD than in subjects without AMD [107]. However, these findings are inconsistent as studies from China [108,109] and India [110], reported that serum SOD levels were increased in patients with AMD. 

What factors account for this discrepancy? Apart from genetic background, one possibility in the observed differences in SOD may be related to differences in diet between the study populations. For example, green tea, which is high in epigallocatechin gallate (EGCG) is consumed in China and curcumin, a bioactive compound in turmeric, is a stable of the Indian diet. EGCG and curcumin stimulate SOD activity [111,112,113,114]. A recent cross-sectional study in China reported that the consumption of green tea, but not black tea, reduced the risk of age-related cataracts [115]. As I previously elucidated, green tea contains significantly higher antioxidants, including EGCG than black tea [75]. Elsewhere, it has been demonstrated that SOD deficiency has been found to be associated with glaucomatous optic neuropathy in human and animal models [116]. Oxidative stress has also been proposed to contribute to retinal ganglion cell (RGC) death in glaucoma [117,118]. Previous studies have also demonstrated that GSH, a tripeptide of glutamate, cysteine, and glycine, has a central role in protecting RGCs against oxidative stress and that glutamate uptake is a rate-limiting step in glial GSH synthesis [118,119]. Consistent with these findings, a reduction in GSH levels has been reported in the plasma of human primary open-angle glaucoma (POAG) patients [120]. Of note, GSH has been found to be decreased in cataractous lenses [121].

### 3.2. The Role of Na+, K+-ATPase Activity in Degenerative Eye Diseases

Previous studies have shown that inhibition of Na+, K+-ATPase activity has been found to accelerate depletion of adenosine triphosphate (ATP), induce mitochondrial depolarization, suppress reactive oxygen species (ROS) scavenging, and enhance ROS production and oxidative stress [122,123,124]. Loss of Na+, K+-ATPase activity is associated with cataract formation [125,126,127,128,129] and age-dependent degeneration in photoreceptors [130]; suggesting a link between loss of Na+, K+-ATPase and AMD. 

### 3.3. Nuclear Factor Erythroid-2-Related Factor 2 Nuclear Factor

Nuclear factor erythroid-2-related factor 2 Nuclear factor (Nrf2) is a key nuclear transcription factor for the systemic antioxidant defence system [131,132]. Inhibition of dysregulation of Nrf2 pathway may contribute to a state of chronic inflammation with a diminished capacity to compensate for conditions of increased oxidative stress [133]. Importantly, Nrf2 is considered as one of the main cellular defence mechanisms against oxidative stresses and ocular diseases including cataracts and AMD [79,134,135,136,137,138,139]. Consistent with this, an animal model for AMD found that Nrf2-deficient mice developed retinal pathology that has similarities with human AMD including deregulated autophagy, oxidative injury and inflammation [139].

### 3.4. Nuclear Factor Kappa-Light-Chain-Enhancer of Activated B Cells (NF-kB)

NF-kB plays a critical role in the expression of inflammatory cytokines, chemokines, immunoreceptors, and cell adhesion molecules that are implicated in the initiation of immune, acute phase, and inflammatory responses [140,141,142,143]. NF-κB is activated in corneal pathologies involving increased plasma levels of LPS and Tumor Necrosis Factor-α (TNF-α), as well as direct UV-B exposure [144]. The constitutive activation of NF- κB has been linked with a wide variety of human diseases including AMD, cataractogenesis and glaucoma [142,145,146,147,148,149].

### 3.5. B-Cell Lymphoma 2 (BCL-2)

Bc1-2 serves an anti-inflammatory function through inhibiting the transcription factor NF-kB [150]. It must be emphasized that reduced Bcl-2 mRNA expression and activity is associated with severe neurodevelopmental disorders such as Down syndrome [151,152] and schizophrenia [153,154]. Hence, this elucidates why the highest prevalence of cataracts are found in individuals with Down syndrome and schizophrenia. In diabetic patients, downregulation of Bcl-2 is associated with a proinflammatory status, enhanced expression of KF-kb, nitric oxide synthase (iNOS) and other inflammatory biomarkers [155]. Hence, downregulation of Bcl-2 in diabetic patients also elucidates why patients with diabetic retinopathy have a higher risk of progressive disease.

### 3.6. Forkhead Box Protein FoxO Proteins

Forkhead box O (FoxO) subfamily of transcription factors regulate expression of target genes involved in DNA damage repair response, apoptosis, metabolism, cellular proliferation, stress tolerance, and longevity [156,157]. Notably, FoxO proteins regulate the expression of intracellular antioxidant enzymes, manganese-superoxide dismutase (SOD) and catalase (CAT) [158,159,160]. In response to oxidative stress, FoxO activity is regulated primarily through regulation of its protein levels, subcellular localization and post-translational modifications. In the aging lens Fox1 and FoxO3a levels are decreased significantly which suggests that age-related down regulation of FoxO1 and FoxO3a expression may contribute to degenerative eye disorders such as cataract formation [161]. 

### 3.7. Interleukin 6 

Interleukin 6 (IL-6) is a proinflammatory cytokine produced by leukocytes, adipocytes, endothelial cells, fibroblasts, and myocytes. IL-6 induces the production of mediators for the release of cytokines such as TNF and IL-1, which drive the inflammatory reaction [162]. IL-6 has been shown to be a key player in chronic low-grade systemic inflammation [163], and IL-6 levels are elevated in inflammatory diseases [164]. The expressions of IL-6 is significantly correlated with the inflammation index in cataract patients [165]. IL-6 levels are increased in schizophrenia [166,167], obesity [168,169,170] and Type 2 diabetes [171,172] which, as previously elucidated, are risk factors for cataracts, AMD and glaucoma.

### 3.8. Paraoxonase 1

Paraoxonase 1 (PON1) plays an essential role in detoxifying the body and reducing oxidative stress [173]. Recently it has been shown that the expression of human PON1 can prevent diabetes development through its antioxidant properties [174,175]. Several studies have reported an association between low PON1 activity and AMD [176,177,178]. Low PON1 activity has also been found to be associated with the pathogenesis of cataracts [179].

## 4. Molecular Mechanisms Underlying Fluoride Contribution to Eye Diseases

Building on the results of these studies, it is necessary to identify the key molecular mechanisms by which chronic F exposure may contribute to degenerative eye diseases. Herein, I identify and investigate some of the key molecular mechanisms by which F exposure contributes to eye diseases as summarized in Table 1. 

### 4.1. Fluoride Inhibition of Carbohydrate Metabolism

Of all the theories advanced to explain the pathogenesis of cataract, the one which seems to have stood the test of time most satisfactorily is that which ascribes the opacification to a defect in the carbohydrate metabolism of the lens [66]. As previously noted, early in vitro studies by Nordmann et al. confirmed that a blockage of the breakdown of sugars by F is followed by cataracts [66]. Further in vitro studies examining metabolism of the lens and of retina have shown that F is an enzyme inhibitor of ocular tissue [67,68,69] and inhibits glycolysis in the retina [70]. Enolase enzymes are known for their role in glucose metabolism [180]. It has been known for many decades that enolase is particularly sensitive to F inhibition [181,182,183]. Furthermore, it is known that the inhibition of enolase results in the formation of advanced glycation end products (AGEs) [184]. AGEs are a significant factor in the pathogenesis of retinopathy and cataracts [185,186]. Consistent with this, recent in vivo studies demonstrated that chronic long-term exposure for six months to F at high and low doses via drinking water significantly increased expression of receptors for advanced glycation end products (RAGE), increased RAGE proteins and increased levels of AGEs in cells. A significant increase in the expression NADPH oxidase 2 (NOX2) was also observed among specimens exposed to fluorine for 6 months. Notably these effects were found to occur at concentrations of just 5 mg/L in drinking water, which is the equivalent to approximately 0.5 mg/L in drinking water for humans. Simultaneous in vitro research with SH-SY5Y cells originating from human neuroblastoma confirmed these results [187]. It is important to note that the NADPH oxidase system participates in generating ROS in the lens [90].

Beyond glucose metabolism, enolase enzymes have been reported to have a number of other non-glycolytic functions, including being a τ-crystallin protein [188], a heat-shock protein [189], hypoxic-stress protein [190], c-Myc binding and transcription protein [191] among others. As F is known to inhibit enolase by binding to active sites within the enolase structure, thereby altering its activity [183] it is therefore plausible that F may alter the activity of non-glycolytic enolase enzymes involved in lens development, repair and protection. For example, crystallins comprise 80–90% of the water-soluble proteins of the transparent lens [192] and are essential determinants of the transparency and refractivity required for lens function [193]. Moreover, τ-crystallin has a distinct function as a lens structural protein [193]. In addition, heat shock proteins are found throughout the various tissues of the eye where they are thought to confer protection from disease states such as cataract, glaucoma, and cancer [194]. Of note, Hsp 40 has also been found to protect the lens from stress induced denaturation [195], while Hsp27 expression is thought to play a role in age-related macular degeneration [196] and cataractogenesis [197]. In addition, variants of Hsp70 have been found to serve as genetic susceptibility factors for susceptibility to cataract and glaucoma in humans [198,199,200]. Moreover, c-Myc binding proteins play an essential role in promoting lens growth and differentiation [201,202] and inactivation of c-myc results in severe eye and lens growth impairment and anterior chamber malformation [202,203]. 

### 4.2. Fluoride and Heat Shock Proteins 

In vivo studies using animal models have found that chronic F exposure can modulate the expression of heat shock proteins in cardiac tissue, liver, kidney and testes [204,205,206]. Recently, Panneerselvam et al. demonstrated that F significantly downregulated the expression of heat shock protein 40 (Hsp40) within living mammalian cells in vivo and upregulated HsP27 and Hsp70 [204]. Consistent with this, Zhao et al. also found that F upregulated mRNA and proteins levels of Hsf27 [205]. Moreover, Chen et al. showed that F exposure significantly increased the expression of Hsp70 in human subjects exposed to F [207]. In this study, the urinary F concentrations in subjects with fluorosis was approximately 2.10 mg/L compared to <1.0 mg/L in controls [207]. Taken together, this data suggests that F has the potential to alter non-glycolytic enolase enzymes activity and protein expression, particularly heat shock proteins that contribute to degenerative eye diseases including cataracts, AMD and glaucoma.

### 4.3. Fluoride Inhibition of Na+, K+-ATPase Activity 

The molecular mechanisms by which F inhibits Na+, K+-ATPase enzyme activity have recently been described [208]. As previously elucidated loss of Na+, K+-ATPase enzyme activity is associated with impaired ROS scavenging, enhanced ROS production [122,123,124], cataract formation [125,126,127,128,129] and age-dependent degeneration in photoreceptors [130]. It is long known that F is an inhibitor of the Na+, K+-ATPase enzyme activity [209,210,211,212,213,214,215,216,217,218,219,220]. However, of fundamental importance, evidence from epidemiological studies confirm this association and provide a biological gradient by which serum F levels may inhibit Na+, K+-ATPase activity in humans. This effect has been found to occur at serum F levels < 5.0 µM in adults [221,222]. Furthermore, it is important to note that AGEs have also been shown to inhibit Na+, K+-ATPase enzyme activity [223]. As previously described, inhibition of enolase leads to the production of AGEs and AGEs are a significant factor in the pathogenesis of retinopathy and cataracts [185,186]. Of interest, inhibition of Na+, K+-ATPase is also a causative factor in the pathogenesis of hypertension [208]. As previously elucidated, hypertension is an established risk factor for the development of cataracts, AMD and glaucoma. Taken together, these findings suggest that one of the key molecular mechanisms by which chronic F exposure may contribute to degenerative eye diseases is via inhibition Na+, K+-ATPase.

### 4.4. Fluoride Inhibition of Nrf2 

Recent in vivo studies have shown that chronic F exposure significantly downregulates mRNA expression of Nrf2 and its downregulatory target genes γ-glutamyl cysteine synthetase (γ-GCS), NAD(P)H quinone dehydrogenase 1 (NQO1) and heme oxygenase 1 (HO-1) [113,224]. It is important to note that the first and rate limiting step in the synthesis of GSH is catalysed by γ-GCS [225] and decreased γ-GCS in turn leads to reduced levels of GSH [226]. As F has been found to downregulate γ-GCS expression, this may elucidate in part one of the molecular mechanisms by which F exposure has been found to decreases GSH activity. Further, HO-1 induction plays an important role in cellular protection against oxidant injury. Overexpression of HO-1 provides protection against inflammation-mediated injury, whereas deficiency in its expression is associated with a chronically inflamed state [227]. In in vivo studies it has been shown that HO-1 protects against rhabdomyolysis (the breakdown of damaged skeletal muscle) and kidney failure. Conversely, inhibition of HO activity exacerbates kidney dysfunction [228,229]. Moreover, HO-1 alleviates ocular surface and corneal inflammation while accelerating wound repair after injury. Wound healing in the cornea is unique because of the need to maintain transparency. The same is true for corneal inflammation, which is intimately linked to the reparative effort [227]. A recent study showed that a deficiency in HO activity, as in HO-2 null mice, exacerbates ocular surface inflammation; increased cell infiltration, expression of inflammatory genes, and production of proinflammatory lipid mediators and impaired wound healing, allowing an acute inflammation to become chronic with the stigma of chronic corneal inflammation such as neovascularization, ulceration, and perforation [230]. 

### 4.5. Fluoride Activation of NF-Kb Expression

Tiwari et al. demonstrated that F inhibits vitamin D receptor (VDR) mRNA expression [231] and low levels of VDR in turn leads to increased levels of NF-kB expression [232]. Consistent with this finding, several in vitro and in vivo studies have demonstrated that F upregulates NF-κB gene expression in in monocytes [233], peripheral blood mononuclear cells [234], macrophages [235,236], as well as kidney [224,225,226,227,228,229,230,231,232,233,234,235,236,237], lung [238], spleen [239] and brain tissue [240,241]. This stimulatory effect has been observed at F concentrations of 2.5 µM [233]. In a study by Misra et al. the authors examined the effect of very low concentrations of beryllium fluorides and found that concentrations as low as 0.002 µM significantly upregulated the activation of NF-κB in macrophages. The effects demonstrated in this study show that beryllium fluorides complexes have much greater cytotoxicity and genotoxicity than other beryllium complexes such as beryllium chloride [242].

### 4.6. Fluoride Downregulates BCL-2, FoxO1 mRNA and Protein Activity and Upregulates IL-6 mRNA Expression and Activity

In in-vivo animal studies chronic exposure to F has consistently been found to significantly reduce Bcl-2 activity induce NF-kB expression and impair antioxidant activity [243,244,245,246,247,248]. Furthermore, numerous studies have demonstrated that F downregulates FoxO1 gene and protein expression resulting in enamel hypomineralization and dental fluorosis [249,250,251,252]. Finally, in regard to IL-6, the seminar research of Liu et al. found that F exposure mediated the expression of over 1000 genes in humans and was found to upregulate specifically the expression of interleukin 6 (IL6) in leucocytes [253]. These findings are in agreement with previous in vitro and in-vivo animal studies which also found that F can induce IL-6 mRNA expression and protein levels [254,255,256,257,258].

### 4.7. Fluoride Inhibits Anitoxidant Activity Including SOD and PON1 Activity

As previously elucidated decreased SOD activity has been reported to be associated with the pathogenesis of cataracts and there is some evidence to suggests it may be associated with AMD in certain populations. In addition, loss of PON1 activity is associated with the pathogenesis of both cataracts and AMD. Sufficient evidence has indicted that consumption of drinking water containing 1ppm F administered to experimental animals contributes to ROS, lipid peroxidation and impaired biological activity of major antioxidant enzymes including SOD, catalase (CAT), and glutathione peroxidase (GPx) [259,260,261,262]. It has been reported that F inhibits production of SOD as a result of the direct action of F ion binding to the enzyme leading to a diminished catalytic activity [263,264,265]. Consistent with these findings, Varol et al. found that the total antioxidant capacity was significantly lower and oxidative stress index significantly higher in subjects (35 males and 44 females; mean age 44.0 + 11.9 years) with a mean urinary F level of 1.91 mg/L compared to healthy controls with a mean urinary F level of 0.49 mg/L [266]. Chen et al. similar found that activity of SOD, CAT and GSH-Px were significantly lower and the concentration of malondialdehyde, a biomarker of oxidative stress and lipid peroxidation, was significantly higher in subjects with fluorosis compared to healthy controls. Notably, the mean SOD level in controls were 86.65 ± 9.20 U/mL compared to 55.56 ± 4.93 U/mL in the high F exposed group. The mean urinary F concentrations in subjects with fluorosis was approximately 2.10 mg/L compared to <1.0 mg/L in controls [207]. These results are consistent with Kalyanalakshmi et al. who also observed enhanced oxidative stress and impaired antioxidant activity in adult male subjects (25–40 years of age) with increasing F exposure [267]. Several other studies have also reported decreases in the activities of SOD, CAT, glutathione-S-transferase (GST), and GPX in humans with increasing F exposure [268,269,270]. Reddy et al. also showed that the activity of malondialdehyde was significantly higher in subjects with fluorosis with mean urinary F levels of 5.94 mg/L compared to controls without fluorosis with mean urinary F levels of 0.41 mg/L [271]. 

Of fundamental importance, a recent cross-sectional study conducted in a F endemic region of India, observed significant increases in lipid peroxidation and protein carbonylation in both serum and crystalline lens of patients with cataracts residing in an endemic fluorosis area compared to controls with cataracts residing in a non-F endemic area. In addition, serum F was significantly increased, and antioxidant activities as measured by SOD and GSG were markedly reduced in patients from the endemic fluorosis area compared to controls. The authors concluded that F ingestion may directly influence cataractogenesis by increased oxidative burden [71]. Furthermore, a recent in vitro study using goats eye lens found that exposure to excessive F resulted in oxidative stress through induced lipid peroxidation and reduced antioxidant activity via reduced GSH, SOD and CAT. On the basis of the results, the authors concluded that uptake of excess consumption of F may be linked with increased oxidative burden which may lead to lens opacification, progression and the development of cataract [65].

#### 4.7.1. Fluoride Inhibits PON1 Activity

Previous investigations have also demonstrated that F exposure significantly inhibits PON-1 activity in humans. The activity of Pon1 was found to decline significantly in a dose dependent manner with increasing serum F concentrations [221]. At 6.8 µM serum F levels the activity of PON1 was found to decline by approximately 30% compared to controls with a serum F level of 3.6 µM. At serum F levels of 14.75 µM Pon1 activity was found to decline by approximately 60% [221]. 

#### 4.7.2. Fluoride Inhibits Glutathione

Previous studies have shown that GSH levels are significantly lower in subjects (19–30 years old) with mild and moderate dental fluorosis compared to healthy controls. Treatment with antioxidant therapy was found to partially restore imbalance of the anti-oxidative defence in patients with fluorosis [272].

## 5. Discussion

The previous sections have described some of the key mechanisms by chronic F exposure can contribute to the pathogenesis of degenerative eyes diseases including cataracts, AMD and glaucoma. In summary, evidence is provided to show that F increases the susceptibility to degenerative eye diseases via multiple pathways and biological interactions. F acts to inhibit enolase, τ-crystallin, Hsp40, Na+, K+-ATPase, Nrf2, γ-GCS, HO-1 Bcl-2, FoxO1, SOD, PON-1 and GSH activity, and upregulates NF-κB, IL-6, AGEs, HsP27 and Hsp70 expression. Moreover, F exposure leads to enhanced oxidative stress and impaired antioxidant activity. Evidence is provided to show that each of these biochemical markers play a role in the pathogenesis of degenerative eye diseases. Collectively, these findings support the hypothesises that chronic F exposure has a causative association in the development and progression of degenerative eye diseases. 

A crucial observation which has emanated from this study, is the explanation as to why among the general population, the prevalence of degenerative eye diseases is highest among individuals with Down syndrome, schizophrenia and diabetes. It is important to note that the association between chronic F exposure and risk of cataracts in Down syndrome was first reported in the 1950s [58,59,60]. The prevalence of diabetes [273,274] and psychiatric disorders [275,276,277,278,279,280] are also significantly higher in people with Down syndrome. Furthermore, the association between schizophrenia and diabetes has been recognized for more than a century [281]. It is known that the prevalence of diabetes is increased 2- to 3-fold in patients with schizophrenia [282,283]. However, to the authors knowledge the mechanisms underlying why the physio pathological features of Down syndrome, schizophrenia and diabetes are associated with higher odds of developing degenerative eye diseases have not been reported previously. 

In the present study, I have elucidated the role of genetics and environmental exposures in degenerative eye diseases, specifically, how aberrant expression of BCL-2 expression is associated with the pathophysiology of Down syndrome, schizophrenia and diabetes. I have elucidated how BCL-2 serves an anti-inflammatory function through inhibiting the transcription factor NF-kB [150] and how activation of NF-kB has been linked to AMD, cataractogenesis and glaucoma. I have further elucidated that F downregulates BCL-2 and induces NF-kB expression. The importance of these observations is self-evident, and in particular elucidates the reason why the burden and prevalence of degenerative eye diseases is significantly higher among individuals with Down syndrome, schizophrenia and diabetes. Taken together, this evidence suggests the possibility that individuals with Down syndrome, schizophrenia and diabetes are genetically predisposed to increased sensitivity to F induced toxicity. However, it is also important to note, that reduced BCL-2 expression is also associated with autism spectrum disorders [284,285,286], which further suggests the possibility that individuals with ASD are a high-risk subgroup for F induced toxicity. 

Aside from the ability of F to alter gene expression and protein activity, F has also been shown to accumulate in human cataract lenses and has previously been reported to be a causative factor in the incidence of senile cataract [61]. A recent cross-sectional study conducted in a F endemic region of India, observed significant increases in lipid peroxidation and protein carbonylation in both serum and crystalline lens of patients with cataracts residing in an endemic fluorosis area. In addition, serum F was significantly increased, and antioxidant activities as measured by SOD and GSG were markedly reduced in patients from the endemic fluorosis area compared to controls. The authors concluded that F ingestion may directly influence cataractogenesis by increased oxidative burden [63]. Macular degeneration and lens opacifying disease have also been observed in workers exposed occupationally to F intoxication [287,288]. It is also known that individuals with lens opacifying disease have an increased risk for AMD compared to those who had no lens opacities [289]. There is also evidence to suggest that chronic F exposure is associated with iridocorneal angle hyperpigmentation and open angle glaucoma [62]. The authors suggested that the trabecular endothelium may be exposed to F toxicity and that heavy trabecular hyperpigmentation appearance may be a feature of endemic fluorosis. It was also reported that the changes underlying the augmentation of trabecular hyperpigmentation observed in subjects may play a role in the development of glaucoma. Interestingly, in this study the mean urinary F level in patients with fluorosis was 2.1 ± 0.60 mg/L compared to 0.38 mg/L in controls [62]. 

Based on these findings, and those mentioned previously demonstrating that F was an inhibitor of enzymes in ocular tissue, further studies examining the possible association between increasing F intake, including water and salt fluoridation and the prevalence of degenerative eye diseases are considered highly desirable. Importantly, the findings of this study, and an understanding of the mechanisms by which F can contribute to degenerative eye diseases, elucidate that certain subgroups of the population may be at increased risk of degenerative eye diseases and suggest that dietary intervention in minimising F intake may reduce the occurrence or severity of AMD, cataracts and glaucoma and related health care burden. 

## 6. Additional Perspectives

The current study has elucidated the role of NF-κB in inflammatory eye diseases and the activation of NF-κB by F. It is important to note that in addition to inflammatory eye diseases, other inflammatory health diseases associated with NF-κB activation include asthma, COPD, atherosclerosis, rheumatoid arthritis, inflammatory bowel disease, diabetes, osteoporosis and cancer [143,149,290,291,292,293,294]. NF-κB activation has also been linked to autism [295,296,297,298], Alzheimer’s disease [299,300,301,302], Parkinson’s disease [303,304] and multiple sclerosis [305,306].

The current study has further elucidated for the first time the role of two heat shock proteins, Hsp27 and Hsp70 in inflammatory eye diseases and the activation by F of their gene expression. In addition to their role in inflammatory eye diseases, activation of Hsp70 gene is implicated in the development of schizophrenia [307,308], autism spectrum disorders [309], asthma [310,311], autoimmune diseases [312,313,314,315], childhood acute lymphoblastic leukaemia [316], breast cancer [317], colon cancer [318], liver cancer [319,320], prostate cancer [321,322], oesophageal cancer [323] and cervical cancer [324]. In addition, recent studies with animal cancer models provided experimental evidence to suggest that Hsp70 is critical for cancer development [325]. Human studies have also shown that overexpression of Hsp27 is associated with several types of human cancer including breast [326,327], ovarian [328], gastric [329], prostate [330], colorectal cancer [331,332], endometrial [333], liver [319], bladder [334], leukaemia, osteosarcoma and lung cancer [335,336]. Moreover, overexpression of Hsp27 in breast, ovarian, gastric, and prostate cancer is associated with aggressive growth and resistance to chemotherapy or radiotherapy, and hence with a poor prognosis [326,327,328,329,330]. There is also considerable evidence indicating that overexpression of Hsp27 enhances tumorigenicity [335]. Furthermore, overexpression of Hsp27 and Hsp70 are implicated in brain tumours [337]. 

Based on these findings, further studies are warranted to examine the association between F intake and the epidemiology of degenerative chronic disorders including neurodegenerative diseases and cancer. 

## 7. Conclusions

In conclusion, this study provides diverse lines of evidence demonstrating that F exposure may contribute to degenerative eye diseases by stimulating or inhibiting biological pathways associated with the pathogenesis of cataract, AMD and glaucoma. As elucidated in this study, F exerts this effect by inhibiting enolase, τ-crystallin, Hsp40, Na+, K+-ATPase, Nrf2, γ-GCS, HO-1 Bcl-2, FoxO1, SOD, PON-1 and GSH activity, and upregulating NF-κB, IL-6, AGEs, HsP27 and Hsp70 expression. Moreover, F exposure leads to enhanced oxidative stress and impaired antioxidant activity. This observation offers another potential relationship to consider when examining the global burden of inflammatory eye diseases including AMD, cataracts and glaucoma worldwide. Based on the evidence presented in this study, it can be concluded that F exposure may be added to the list of identifiable risk factors associated with pathogenesis of degenerative eye diseases. The broader impact of these findings suggests that modifying or reducing F intake may lead to an overall reduction in the modifiable risk factors associated with degenerative eye diseases particularly among persons with Down syndrome, schizophrenia and diabetes. Further studies are required to examine this association and determine differences in prevalence rates amongst fluoridated and non-fluoridated communities, taking into consideration other dietary sources of F such as tea. Finally, the findings of this study elucidate molecular pathways sensitive to F exposure that may suggest a possible association between F exposure and other inflammatory diseases including, pulmonary diseases, neurodegenerative diseases, neurodevelopmental disorders and cancer. Further studies are also warranted to examine these associations. 

## Figures and Tables

**Table 1:** Summary of molecular mechanisms by which fluoride contributes to eye diseases.

Factor	Effect of F	Contribution to Degenerative Eye Diseases
Enolase	↓	Loss of enolase induces cataractogenesis. τ-Crystallin, heat shock proteins, hypoxic stress proteins and c-Myc binding proteins possess enolase activity. These proteins are essential for lens function repair and protection.
Heat Shock Proteins		
Hsp 40	↓	Hsp 40 has been found to protect the lens from stress induced denaturation.
Hsp 27	↑	Hsp27 expression associated with AMD and cataracts.
Hsp 70	↑	Hsp70 expression associated with increased risk of cataracts and glaucoma
FoxO proteins	↓	FoxO proteins regulate antioxidant enzymes. Down regulation of FoxO1 and FoxO3a expression contributes to degenerative eye disorders such as cataract formation.
Na+, K+-ATPase	↓	Inhibition of Na+, K+-ATPase leads to enhanced ROS production and oxidative stress. Loss of Na+, K+-ATPase associated with cataractogenesis and age-dependent degeneration in photoreceptors, suggesting a link between loss of Na+, K+-ATPase and AMD. Loss of Na+, K+-ATPase linked to hypertension. Hypertension is a risk factor for cataracts, AMD and glaucoma.
PON1	↓	PON1 is an antioxidant and reduces oxidative stress. Low PON1 activity associated with AMD and cataracts.
IL-6	↑	IL-6 has been shown to be a key player in chronic low-grade systemic inflammation. Associated with cataracts, AMD and glaucoma.
Nrf2	↓	Inhibition of dysregulation of Nrf2 pathway contributes to a state of chronic systemic inflammation with a diminished capacity to compensate for conditions of increased oxidative stress. Loss of Nrf2 is associated with AMD.
NF-kB	↑	NF-kB plays a critical role in the expression of inflammatory cytokines. Expression of NF-kB linked to AMD, cataracts and glaucoma.
BCL-2	↓	Has anti-inflammatory properties, reduced expression associated with pathological states and degenerative eye diseases.
Antioxidants	↓	Impaired antioxidant activity leads to oxidative stress. Oxidative stress strongly associated with AMD, cataracts and glaucoma.

Table 1 summarises key molecular pathways by which F contributes to eye diseases. Hsp: Heat shock protein; FoxO: Foxhead box ‘Other’ proteins; PON1: Paraoxonase 1; N2f2: Nuclear factor erythroid-2-related factor 2 Nuclear factor; IL6: Interleukin 6; NF-Kb: Nuclear Factor kappa-light-chain-enhancer of activated B cells; BCL-2: B-cell lymphoma 2.

## References

[1] Wang G.F., Zou X.L. (2012). Tissue factor with age-related macular degeneration. Int. J. Ophthalmol..

[2] Cai L., Liao H.F., Zhang X.J., Shao Y., Xu M., Yi J.L. (2013). Acetylcholinesterase function in apoptotic retina pigment epithelial cells induced by H_2_O_2_. Int. J. Ophthalmol..

[3] Chiu C.J., Chang M.L., Zhang F.F., Li T., Gensler G., Schleicher M., Taylor A. (2014). The relationship of major American dietary patterns to age-related macular degeneration. Am. J. Ophthalmol..

[4] Zhang Q.Y., Tie L.J., Wu S.S., Lv P.L., Huang H.W., Wang W.Q., Wang H., Ma L. (2016). Overweight, Obesity, and Risk of Age-Related Macular Degeneration. Investig. Ophthalmol. Vis. Sci..

[5] Hyman L., Schachat A.P., He Q., Leske M.C. (2000). Hypertension, cardiovascular disease, and age-related macular degeneration. Age-Related Macular Degeneration Risk Factors Study Group. Arch. Ophthalmol..

[6] Tan J.S., Mitchell P., Smith W., Wang J.J. (2007). Cardiovascular risk factors and the long-term incidence of age-related macular degeneration: The Blue Mountains Eye Study. Ophthalmology.

[7] Tomany S.C., Wang J.J., Van Leeuwen R., Klein R., Mitchell P., Vingerling J.R., Klein B.E., Smith W., De Jong P.T. (2004). Risk factors for incident age-related macular degeneration: Pooled findings from 3 continents. Ophthalmology.

[8] Chen X., Rong S.S., Xu Q., Tang F.Y., Liu Y., Gu H., Tam P.O., Chen L.J., Brelén M.E., Pang C.P. (2014). Diabetes mellitus and risk of age-related macular degeneration: A systematic review and meta-analysis. PLoS ONE.

[9] He M.S., Chang F.L., Lin H.Z., Wu J.L., Hsieh T.C., Lee Y.C. (2018). The Association Between Diabetes and Age-Related Macular Degeneration Among the Elderly in Taiwan. Diabetes Care.

[10] Evans J.R. (2001). Risk factors for age-related macular degeneration. Prog. Retin. Eye Res..

[11] Babizhayev M.A., Yegorov Y.E. (2016). Reactive Oxygen Species and the Aging Eye: Specific Role of Metabolically Active Mitochondria in Maintaining Lens Function and in the Initiation of the Oxidation-Induced Maturity Onset Cataract—A Novel Platform of Mitochondria-Targeted Antioxidants with Broad Therapeutic Potential for Redox Regulation and Detoxification of Oxidants in Eye Diseases. Am. J. Ther..

[12] Prokofyeva E., Wegener A., Zrenner E. (2013). Cataract prevalence and prevention in Europe: A literature review. Acta Ophthalmol..

[13] Tavani A., Negri E., La Vecchia C. (1996). Food and nutrient intake and risk of cataract. Ann. Epidemiol..

[14] Abraham A.G., Condon N.G., West Gower E. (2006). The new epidemiology of cataract. Ophthalmol. Clin. N. Am..

[15] Trautner C., Haastert B., Richter B., Berger M., Giani G. (2003). Incidence of Blindness in Southern Germany Due to Glaucoma and Degenerative Conditions. Investig. Ophthalmol. Vis. Sci..

[16] Yu X., Lyu D., Dong X., He J., Yao K. (2014). Hypertension and risk of cataract: A meta-analysis. PLoS ONE.

[17] Gnad H.D., Rett A. (1979). Ophthalmologische symptome beim Down syndrom. Wien. Klin. Wochenschr..

[18] Rochels R., Nover A., Schmid F. (1977). Ophthalmologische symptome beim Mongolismussyndrom. Albrecht Von Graefes. Arch. Klin. Exp. Ophthalmol..

[19] Da Cunha R.P., Moreira J.B. (1996). Ocular findings in Down’s syndrome. Am. J. Ophthalmol..

[20] Puri B.K., Singh I. (2007). Prevalence of cataract in adult Down’s syndrome patients aged 28 to 83 years. Clin. Pract. Epidemiol. Ment. Health.

[21] Ruigomez A., Garcia Rodriguez L.A., Dev V.J., Arellano F., Raniwala J. (2000). Are schizophrenia or antipsychotic drugs a risk factor for cataracts?. Epidemiology.

[22] Li L., Wan X.H., Zhao G.H. (2014). Meta-analysis of the risk of cataract in type 2 diabetes. BMC Ophthalmol..

[23] Khairallah M., Kahloun R., Bourne R., Limburg H., Flaxman S.R., Jonas J.B., Keeffe J., Leasher J., Naidoo K., Pesudovs K. (2015). Number of People Blind or Visually Impaired by Cataract Worldwide and in World Regions, 1990 to 2010. Investig. Ophthalmol. Vis. Sci..

[24] McMonnies C.W. (2016). Glaucoma history and risk factors. J. Optom..

[25] Hirooka K., Shiraga F. (2007). Potential role for angiotensin-converting enzyme inhibitors in the treatment of glaucoma. Clin. Ophthalmol..

[26] Deloitte Access Economics The Economic Impact of Vision Impairment and Blindness in the Republic of Ireland NCBI (National Council for the Blind of Ireland) May 2011. http://www.eyedoctors.ie/documents/Cost_of_Sight_Loss_Full_Repor.pdf.

[27] Eurostat. https://ec.europa.eu/eurostat/statistics-explained/index.php?title=File:Surgical_operations_and_procedures_performed_in_hospitals_%E2%80%94_top_10_procedures_group_1,_2015_or_2016_(per_100_000_inhabitants)_HLTH18.png.

[28] Akuffo K.O., Nolan J., Stack J., Moran R., Feeney J., Kenny R.A., Peto T., Dooley C., O’Halloran A.M., Cronin H. (2015). Prevalence of age-related macular degeneration in the Republic of Ireland. Br. J. Ophthalmol..

[29] Nolan J., Kenny R., O’Regan C., Cronin H., Loughman J., Conolly E., Kearney P., Beatty S. (2010). Macular Pigment Optical Density in an Aging Irish Population: The Irish Longitudinal Study on Ageing. Ophthalm. Res..

[30] Augood C.A., Vingerling J.R., de Jong P.T., Chakravarthy U., Seland J., Soubrane G., Tomazzoli L., Topouzis F., Bentham G., Rahu M. (2006). Prevalence of age-related maculopathy in older Europeans: The European Eye Study (EUREYE). Arch. Ophthalmol..

[31] Colijn J.M., Buitendijk G.H.S., Prokofyeva E., Alves D., Cachulo M.L., Khawaja A.P., Cougnard-Gregoire A., Merle B.M.J., Korb C., Erke M.G. (2017). Prevalence of Age-Related Macular Degeneration in Europe: The Past and the Future. Ophthalmology.

[32] Owen C.G., Jarrar Z., Wormald R., Cook D.G., Fletcher A.E., Rudnicka A.R. (2012). The estimated prevalence and incidence of late stage age related macular degeneration in the UK. Br. J. Ophthalmol..

[33] Jonasson F., Arnarsson A., Sasaki H., Peto T., Sasaki K., Bird A.C. (2003). The prevalence of age-related maculopathy in iceland: Reykjavik eye study. Arch. Ophthalmol..

[34] Björnsson O.M., Syrdalen P., Bird A.C., Peto T., Kinge B. (2006). The prevalence of age-related maculopathy (ARM) in an urban Norwegian population: The Oslo Macular study. Acta Ophthalmol. Scand..

[35] Klein R., Klein B.E.K., Cruickshanks K.J. (1999). The prevalence of age-related maculopathy by geographic region and ethnicity. Prog. Retin. Eye Res..

[36] Kawasaki R., Wang J.J., Ji G.J., Taylor B., Oizumi T., Daimon M., Kato T., Kawata S., Kayama T., Tano Y. (2008). Prevalence and risk factors for agerelated macular degeneration in an adult Japanese population: The Funagata study. Ophthalmology.

[37] Klein R., Chou C.F., Klein B.E., Zhang X., Meuer S.M., Saaddine J.B. (2011). Prevalence of age-related macular degeneration in the US population. Arch. Ophthalmol..

[38] Klein R., Rowland M.L., Harris M.I. (1995). Racial/ethnic differences in age-related maculopathy: Third National Health and Nutrition Survey. Ophthalmology.

[39] Congdon N., Vingerling J.R., Klein B.E., West S., Friedman D.S., Kempen J., O’Colmain B., Wu S.Y., Taylor H.R., Eye Diseases Prevalence Research Group (2004). Prevalence of Cataract and Pseudophakia/Aphakia Among Adults in the United States. Arch. Ophthalmol..

[40] Lindstrom R. (2015). Thoughts on cataract surgery. Rev. Ophthalmol..

[41] Busbee B.G., Brown M.M., Brown G.C., Sharma S. (2002). Incremental cost-effectiveness of initial cataract surgery. Ophthalmology.

[42] American Diabetes Association (2013). Economic costs of diabetes in the U.S. in 2012. Diabetes Care.

[43] Keel S., Xie J., Foreman J., van Wijngaarden P., Taylor H.R., Dirani M. (2017). Prevalence of Age-Related Macular Degeneration in Australia: The Australian National Eye Health Survey. JAMA Ophthalmol..

[44] Keeffe J.E., Taylor H.R. (1996). Cataract surgery in Australia 1985–1994. Aust. N. Z. J. Ophthalmol..

[45] Tan A.G., Wang J.J., Rochtchina E., Mitchell P. (2006). Comparison of age-specific cataract prevalence in two population-based surveys 6 years apart. BMC Ophthalmol..

[46] McCarty C.A., Mukesh B.N., Fu C.L., Taylor H.R. (1999). The epidemiology of cataract in Australia. Am. J. Ophthalmol..

[47] Rochtchina E., Mukesh B.N., Wang J.J., McCarty C.A., Taylor H.R., Mitchell P. (2003). Projected prevalence of age-related cataract and cataract surgery in Australia for the years 2001 and 2021: Pooled data from two population-based surveys. Clin. Exp. Ophthalmol..

[48] Worsley D., Worsley A. (2015). Prevalence predictions for age-related macular degeneration in New Zealand have implications for provision of healthcare services. N. Z. Med. J..

[49] Access Economics (2010). Clear focus—The Economic Impact of Vision Loss in New Zealand in 2009. A Report for Vision 2020 Australia in Support of the Vision 2020 New Zealand Trust.

[50] National Health Committee (2015). Age-Related Macular Degeneration, Tier 2 Assessment Consultation Submissions.

[51] Blind Foundation Eye Diseases. https://blindfoundation.org.nz/eye-info/latest-statistics/.

[52] Sorsby A., Harding R. (1960). Experimental degeneration of the retina. Br. J. Ophthalmol..

[53] Sorsby A., Harding R. (1966). Oxidizing agents as potentiators of the retinotoxic action of sodium fluoride, sodium iodate and sodium iodoacetate. Nature.

[54] Orzalesi N., Grignolo A., Calabria A. (1967). Experimental degeneration of the rabbit retina induced by sodium fluoride. Exp. Eye Res..

[55] Orzalesi N., Grignolo A., Calabria G.A., Castellazzo R. (1967). A study on the fine structure and the rhodopsin cycle of the rabbit retina in experimental degeneration induced by diaminodiphenoxypentane. Exp. Eye Res..

[56] Vanysek J., Anton M., Hrachovina V., Moster M. (1969). Some metabolic disturbances of the retina due to the effect of natrium fluoride. Ophthalmologica.

[57] Shukla N., Pandey G.S. (1991). Fluoride level in cataract lenses in an urban area of India. Fluoride.

[58] Rapaport I. (1957). Les opacifications du cristallin mongolisme et cataracte sénile (Donées statisques récentes). Rev. Anthropol. (Paris).

[59] Rapaport I. (1957). Contribution a l’étude etiologique du mongolisme: Rôle des inhibiteurs enzymatiques. L’Encéphale.

[60] Rapaport I. (1960). Oligophrénie mongolienne et ectodermoses congénitales. Ann. Dermatol. Syphiligr..

[61] Kas’ianenko A.S., Korneva T.S., Kovgan N.I. (1984). Dissemination of senile cataract among the population of Poltava Province consuming water with various fluorine levels. Oftalmol. Zh..

[62] Aytuluner E., Mensiz E. (2002). Heavy iridocorneal angle hyperpigmentation and glaucoma associated with fluorosis. J. Toxicol. Cutaneous Ocular Toxicol..

[63] Tomar S., Sharma A., Tripathi S. Fluoride intake increases oxidative burden of cataractogenesis in fluoride endemic areas in India, Abstract. Proceedings of the XXXII Congress of the ESCRS.

[64] Aytuluner E., Mensiz E., Candir O., Aydin S. (2003). Cataractogenic effect of fluorosis in an animal model. J. Toxicol. Cutaneous Ocular Toxicol..

[65] Mishra S., Tomar S., Sharma A., Chauhan D.S., Tripathi S. (2014). Fluoride Induces Morphological and Biochemical Changes in Goat Eye Lens. J. Environ. Anal. Toxicol..

[66] Nordmann J., Mandel P., Archard M. (1954). Inhibition of sugar metabolism in the lens. Br. J. Ophthal..

[67] Ashton N., Graymore C., Petlar C. (1957). Studies on developing retinal vessels. Br. J. Ophthalmol..

[68] Graymore C. (1959). In vitro swelling of the kitten retina induced by sodium fluoride inhibition. Br. J. Ophthalmol..

[69] Kleifeld O., Hockwine O., Ayberk N. (1956). The effect of sodium fluoride on the metabolism of the lens. Graefes Arch. Ophthalmol..

[70] Dickens F., Simer F. (1929). Observations on Tissue Glycolysis: The effect of fluoride and some other substances. Biochem. J..

[71] Tomar S. (2014). Fluoride intake increases oxidative burden of cataractognesis in fluoride endemic areas in India. J. Clin. Exp. Ophthalmol..

[72] (2001). Recommendations for using fluoride to prevent and control dental caries in the United States. MMWR.

[73] Chan L., Mehra A., Saikat S., Lynch P. (2013). Human exposure assessment of fluoride from tea (Camellia sinensis L.). Food Res. Int..

[74] Waugh D.T., Potter W., Limeback H., Godfrey M. (2016). Risk assessment of fluoride intake from tea in the republic of Ireland and its implications for public health and water fluoridation. Int. J. Environ. Res. Public Health.

[75] Waugh D.T., Godfrey M., Limeback H., Potter W. (2017). Black Tea Source, Production, and Consumption: Assessment of Health Risks of Fluoride Intake in New Zealand. J. Environ. Public Health.

[76] (2013). Scientific Opinion on Dietary Reference Values for fluoride. EFSA J..

[77] Water Fluoridation for the Prevention of Dental Caries (Review). The Cochrane Library 2015, Issue 6. https://www.cochrane.org/CD010856/ORAL_water-fluoridation-prevent-tooth-decay.

[78] (2003). Opinion of the Scientific Committee on Cosmetic Products and Non-Food Products Intended for Consumers Concerning the Safety of Fluorine Compounds in Oral Hygiene Products for Children Under the Age of 6 Years. https://ec.europa.eu/health/ph_risk/committees/04_sccp/docs/sccp_o_024.pdf.

[79] Liu X.F., Zhou D.D., Xie T., Hao J.L., Malik T.H., Lu C.B., Qi J., Pant O.P., Lu C.W. (2018). The Nrf2 Signaling in Retinal Ganglion Cells under Oxidative Stress in Ocular Neurodegenerative Diseases. Int. J. Biol. Sci..

[80] Dröge W. (2002). Free radicals in the physiological control of cell function. Physiol. Rev..

[81] Grune T., Sommerburg O., Siems W.G. (2000). Oxidative stress in anemia. Clin. Nephrol..

[82] Spector A. (1995). Oxidative stress-induced cataract: Mechanism of action. FASEB J..

[83] Alvarado J.A., Murphy C.G., Polansky J.R., Juster R. (1981). Age-related changes in human trabecular meshwork cellularity. Investig. Ophthalmol. Vis. Sci..

[84] Kahn M.G., Giblin F.J., Epstein D.L. (1983). Glutathione in calf trabecular meshwork and its relation to aqueous humor outflow facility. Investig. Ophthalmol. Vis. Sci..

[85] Young R.W. (1988). Solar radiation and age-related macular degeneration. Surv. Ophthalmol..

[86] Kowluru R.A., Kern T.S., Engerman R.L., Armstrong D. (1996). Abnormalities of retinal metabolism in diabetes or experimental galactosemia. III. Effects of antioxidants. Diabetes.

[87] Rao N.A., Wu G.S. (2000). Free radical mediated photoreceptor damage in uveitis. Prog. Retin. Eye Res..

[88] Hedge K.R., Varma S.D. (2005). Prevention of cataract by pyruvate in experimentally diabetic mice. Mol. Cell Biochem..

[89] Hegde K.R., Kovtun S., Varma S.D. (2010). Inhibition of glycolysis in the retina by oxidative stress: Prevention by pyruvate. Mol. Cell. Biochem..

[90] Berthoud V.M., Beyer E.C. (2009). Oxidative stress, lens gap junctions, and cataracts. Antioxid. Redox Signal..

[91] Izzotti A., Bagnis A., Saccà S.C. (2006). The role of oxidative stress in glaucoma. Mutat. Res..

[92] McMonnies C.W. (2017). Reactive oxygen species, oxidative stress, glaucoma and hyperbaric oxygen therapy. J. Optom..

[93] Babizhayev M.A., Deyev A.I., Yermakova V.N., Brikman I.V., Bours J. (2004). Lipid peroxidation and cataracts: N-acetylcarnosine as a therapeutic tool to manage age-related cataracts in human and in canine eyes. Drugs R&D.

[94] Brennan L.A., Kantorow M. (2009). Mitochondrial function and redox control in the aging eye: Role of MsrA and other repair systems in cataract and macular degenerations. Exp. Eye Res..

[95] Ottonello S., Foroni C., Carta A., Petrucco S., Maraini G. (2000). Oxidative stress and age-related cataract. Ophthalmologica..

[96] Beswick H.T., Harding J.J. (1984). Conformational changes induced in bovine lens alpha-crystallin by carbamylation. Relevance to cataract. Biochem. J..

[97] Fukai T., Ushio-Fukai M. (2011). Superoxide dismutases: Role in redox signaling, vascular function, and diseases. Antioxid. Redox Signal..

[98] Reddy V.N. (1990). Glutathione and its function in the lens-an overview. Exp. Eye Res..

[99] Rhee S.G., Kang S.W., Chang T.S., Jeong W., Kim K. (2001). Peroxiredoxin, a novel family of peroxidases. IUBMB Life.

[100] Spector A., Ma W., Wang R.R., Kleiman N.J. (1997). Microperoxidases catalytically degrade reactive oxygen species and may be anti-cataract agents. Exp. Eye Res..

[101] Forman H.J., Zhang H., Rinna A. (2008). Glutathione: Overview of its protective roles, measurement, and biosynthesis. Mol. Aspects Med..

[102] Jarrett S.G., Boulton M.E. (2012). Consequences of oxidative stress in age-related macular degeneration. Mol. Aspects Med..

[103] Maurya O.P., Mohanty L., Bhaduri G., Chandra A. (2006). Role of anti-oxidant enzymes superoxide dismutase and catalase in the development of cataract: Study of serum levels in patients with senile and diabetic cataracts. J. Indian Med. Assoc..

[104] Ozmen B., Ozmen D., Erkin E., Güner I., Habif S., Bayindir O. (2002). Lens superoxide dismutase and catalase activities in diabetic cataract. Clin. Biochem..

[105] Babizhayev M.A., Deyev A.I., Linberg L.F. (1988). Lipid peroxidation as a possible cause of cataract. Mech. Age Dev..

[106] Obara Y. (1995). The oxidative stress in the cataract formation. Nippon-Ganka-Gakkai-Zasshi.

[107] Yildirim Z., Ucgun N.I., Yildirim F. (2011). The role of oxidative stress and antioxidants in the pathogenesis of age-related macular degeneration. Clinics.

[108] Shen X.L., Jia J.H., Zhao P., Fan R., Pan X.Y., Yang H.M., Liu L. (2012). Changes in blood oxidative and antioxidant parameters in a group of Chinese patients with age-related macular degeneration. J. Nutr. Health Aging.

[109] Jia L., Dong Y., Yang H., Pan X., Fan R., Zhai L. (2011). Serum superoxide dismutase and malondialdehyde levels in a group of Chinese patients with age-related macular degeneration. Aging Clin. Exp. Res..

[110] Anand A., Sharma N.K., Gupta A., Prabhakar S., Sharma S.K., Singh R. (2013). Superoxide dismutase1 levels in North Indian population with age-related macular degeneration. Oxid. Med. Cell. Longev..

[111] Thangapandiyan S., Miltonprabu S. (2014). Epigallocatechin gallate supplementation protects against renal injury induced by fluoride intoxication in rats: Role of Nrf2/HO-1 signaling. Toxicol. Rep..

[112] Shen L.R., Xiao F., Yuan P., Chen Y., Gao Q.K., Parnell L.D., Meydani M., Ordovas J.M., Li D., Lai C.Q. (2012). Curcumin-supplemented diets increase superoxide dismutase activity and mean lifespan in Drosophila. Age (Dordr.).

[113] Akinyemi A.J., Oboh G., Ogunsuyi O., Abolaji A.O., Udofia A. (2018). Curcumin-supplemented diets improve antioxidant enzymes and alter acetylcholinesterase genes expression level in Drosophila melanogaster model. Metab. Brain Dis..

[114] Nabavi S.F., Nabavi S.M., Abolhasani F., Moghaddam A.H., Eslami S. (2012). Cytoprotective effects of curcumin on sodium fluoride-induced intoxication in rat erythrocytes. Bull. Environ. Contam. Toxicol..

[115] Sheng Y., He F., Lin J.F., Shen W., Qiu Y.W. (2016). Tea and Risk of Age-Related Cataracts: A Cross-Sectional Study in Zhejiang Province, China. J. Epidemiol..

[116] Yuki K., Ozawa Y., Yoshida T., Kurihara T., Hirasawa M., Ozeki N., Shiba D., Noda K., Ishida S., Tsubota K. (2011). Retinal Ganglion Cell Loss in Superoxide Dismutase 1 Deficiency. Investig. Ophthalmol. Vis. Sci..

[117] Klein J.A., Longo-Guess C.M., Rossmann M.P., Seburn K.L., Hurd R.E., Frankel W.N., Bronson R.T., Ackerman S.L. (2002). The harlequin mouse mutation downregulates apoptosis-inducing factor. Nature.

[118] Tezel G. (2006). Oxidative stress in glaucomatous neurodegeneration: Mechanisms and consequences. Prog. Retin. Eye Res..

[119] Schulz J.B., Lindenau J., Seyfried J., Dichgans J. (2000). Glutathione, oxidative stress and neurodegeneration. Eur. J. Biochem..

[120] Gherghel D., Griffiths H.R., Hilton E.J., Cunliffe I.A., Hosking S.L. (2005). Systemic reduction in glutathione levels occurs in patients with primary open-angle glaucoma. Investig. Ophthalmol. Vis. Sci..

[121] Spector A. (1984). Oxidation and cataract. Ciba Found Symp..

[122] Li Q., Pogwizd S.M., Prabhu S.D., Zhou L. (2014). Inhibiting Na+/K+ ATPase can impair mitochondrial energetics and induce abnormal Ca2+ cycling and automaticity in guinea pig cardiomyocytes. PLoS ONE.

[123] Roy S., Dasgupta A., Banerjee U., Chowdhury P., Mukhopadhyay A., Saha G., Singh O. (2016). Role of membrane cholesterol and lipid peroxidation in regulating the Na+/K+-ATPase activity in schizophrenia. Indian J. Psychiatry.

[124] Yan Y., Haller S., Shapiro A., Malhotra N., Tian J., Xie Z., Malhotra D., Shapiro J.I., Liu J. (2012). Ouabain-stimulated trafficking regulation of the Na/K-ATPase and NHE3 in renal proximal tubule cells. Mol. Cell. Biochem..

[125] Delamere N.A., Tamiya S. (2004). Expression, regulation and function of Na,K-ATPase in the lens. Prog. Retin. Eye Res..

[126] Unakar N.J., Tsui J., Johnson M. (1997). Effect of pretreatment of germanium-1322 on NaC,KC-ATPase and galactose cataracts. Curr. Eye Res..

[127] Yokoyama T., Sasaki H., Giblin F.J., Reddy V.N. (1994). A physiological level of ascorbate inhibits galactose cataract in guinea pigs by decreasing polyol accumulation in the lens epithelium: A dehydroascorbate-linked mechanism. Exp. Eye Res..

[128] Ahmad S.S., Tsou K.C., Ahmad S.I., Rahman M.A., Kirmani T.H. (1985). Studies on cataractogenesis in humans and rats with Alloxan-induced diabetes. Ophthalm. Res..

[129] Mizuno G.R., Chapman C.J., Chipault J.R., Pfeiffer D.R. (1981). Lipid composition and(NaC C KC)-ATPase activity in rat lens during triparanol-induced cataract formation. Biochim. Biophys. Acta.

[130] Luan Z., Reddig K., Li H.S. (2014). Loss of Na+/K+-ATPase in Drosophila photoreceptors leads to blindness and age-dependent neurodegeneration. Exp. Neurol..

[131] Li Z., Dong X., Liu H., Chen X., Shi H., Fan Y., Hou D., Zhang X. (2013). Astaxanthin protects ARPE-19 cells from oxidative stress via upregulation of Nrf2-regulated phase II enzymes through activation of PI3K/Akt. Mol. Vis..

[132] Rushworth S.A., Chen X.L., Mackman N., Ogborne R.M., O’Connell M.A. (2005). Lipopolysaccharide-induced heme oxygenase-1 expression in human monocytic cells is mediated via Nrf2 and protein kinase C. J. Immunol..

[133] Pickering A.M., Linder R.A., Zhang H., Forman H.J., Davies K.J. (2012). Nrf2-dependent induction of proteasome and Pa28αβ regulator are required for adaptation to oxidative stress. J. Biol. Chem..

[134] Liu X.F., Hao J.L., Xie T., Malik T.H., Lu C.B., Liu C., Shu C., Lu C.W., Zhou D.D. (2017). Nrf2 as a target for prevention of age-related and diabetic cataracts by against oxidative stress. Aging Cell..

[135] Zhou T., Zong R., Zhang Z., Zhu C., Pan F., Xiao X., Liu Z., He H., Ma J.X., Liu Z. (2012). SERPINA3K protects against oxidative stress via modulating ROS generation/degradation and KEAP1-NRF2 pathway in the corneal epithelium. Investig. Ophthalmol. Vis. Sci..

[136] Lambros M.L., Plafker S.M. (2016). Oxidative Stress and the Nrf2 Anti-Oxidant Transcription Factor in Age-Related Macular Degeneration. Adv. Exp. Med. Biol..

[137] Cao Y., Wang L., Zhao J., Zhang H., Tian Y., Liang H., Ma Q. (2016). Serum Response Factor Protects Retinal Ganglion Cells Against High-Glucose Damage. J. Mol. Neurosci..

[138] Cho H., Hartsock M.J., Xu Z., He M., Duh E.J. (2015). Monomethyl fumarate promotes Nrf2-dependent neuroprotection in retinal ischemia-reperfusion. J. Neuroinflamm..

[139] Zhao Z., Chen Y., Wang J., Sternberg P., Freeman M.L., Grossniklaus H.E., Cai J. (2011). Age-Related Retinopathy in NRF2-Deficient Mice. PLoS ONE.

[140] Jobin C., Bradham C.A., Russo M.P., Juma B., Narula A.S., Brenner D.A., Sartor R.B. (1999). Curcumin blocks cytokine-mediated NF-kappa B activation and proinflammatory gene expression by inhibiting inhibitory factor I-kappa B kinase activity. J. Immunol..

[141] Baeuerle P.A., Henkle T. (1994). Function and activation of NF-κB in the immune system. Annu. Rev. Immunol..

[142] Barnes P.J., Karin M. (1997). Nuclear factor-κB, a pivotal transcription factor in chronic inflammatory diseases. N. Engl. J. Med..

[143] Pahl H.L. (1999). Activators and target genes of Rel/NF-kappaB transcription factors. Oncogene.

[144] Alexander G., Carlsen H., Blomhoff R. (2006). Corneal NF-κB activity is necessary for the retention of transparency in the cornea of UV-B-exposed transgenic reporter mice. Exp. Eye Res..

[145] Jin X.H., Ohgami K., Shiratori K., Koyama Y., Yoshida K., Kase S., Ohno S. (2006). Inhibition of nuclear factor-kappa B activation attenuates hydrogen peroxide-induced cytotoxicity in human lens epithelial cells. Br. J. Ophthalmol..

[146] Kauppinen A., Paterno J.J., Blasiak J., Salminen A., Kaarniranta K. (2016). Inflammation and its role in age-related macular degeneration. Cell. Mol. Life Sci..

[147] Lukiw W., Jones B., Bhattacharjee S., Alexandrov P., Dua P., Zhao Y. (2013). TREM2 (chr6p21.1) and CFH (chr1q32) regulation by NF-kB-sensitive miRNAs in age-related macular degeneration (AMD) and Alzheimer’s disease (AD). Investig. Ophthalmol. Vis. Sci..

[148] Lupien C., Horner P., Calkins D. (2013). NFkB signaling in retinal glia mediates progressive neural degeneration and vision decline in glaucoma. Investig. Ophthalmol. Vis. Sci..

[149] Ghosh S., Shang P., Yazdankhah M., Bhutto I., Hose S., Montezuma S.R., Luo T., Chattopadhyay S., Qian J., Lutty G.A. (2017). Activating the AKT2-nuclear factor-κB-lipocalin-2 axis elicits an inflammatory response in age-related macular degeneration. J. Pathol..

[150] Grimm S., Bauer M.K.A., Baeuerle P.A., Schulze-Osthoff K. (1996). Bcl-2 down-regulates the activity of transcription factor NF-kB induced upon apoptosis. J. Cell. Biol..

[151] Seidl R., Bidmon B., Bajo M., Yoo P.C., Cairns N., LaCasse E.C., Lubec G. (2001). Evidence for apoptosis in the fetal Down syndrome brain. J. Child Neurol..

[152] Wolvetang E.J., Wilson T.J., Sanij E., Busciglio J., Hatzistavrou T., Seth A., Hertzog P.J., Kola I. (2003). ETS2 overexpression in transgenic models and in Down syndrome predisposes to apoptosis via the p53 pathway. Hum. Mol. Genet..

[153] Zampieri B.L., Biselli-Périco J.M., de Souza J.E., Bürger M.C., Silva Júnior W.A., Goloni-Bertollo E.M., Pavarino E.C. (2014). Altered Expression of Immune-Related Genes in Children with Down Syndrome. PLoS ONE.

[154] Fredrik Jarskog L., Gilmore J.H., Selinger E.S., Lieberman J.A. (2000). Cortical Bcl-2 Protein Expression and Apoptotic Regulation in Schizophrenia. Biol. Psychiatry.

[155] Cipollone F., Chiarelli F., Iezzi A., Fazia M.L., Cuccurullo C., Pini B., De Cesare D., Torello M., Tumini S., Cuccurullo F. (2005). Relationship between reduced BCL-2 expression in circulating mononuclear cells and early nephropathy in type 1 diabetes. Int. J. Immunopathol. Pharmacol..

[156] Calnan D.R., Brunet A. (2008). The FoxO code. Oncogene.

[157] Van der Horst A., Burgering B.M. (2007). Stressing the role of FoxO proteins in lifespan and disease. Nat. Rev. Mol. Cell. Biol..

[158] Kops G.J., Dansen T.B., Polderman P.E., Saarloos I., Wirtz K.W., Coffer P.J., Huang T.T., Bos J.L., Medema R.H., Burgering B.M.T. (2002). Forkhead transcription factor FOXO3a protects quiescent cells from oxidative stress. Nature.

[159] Nemoto S., Finkel T. (2002). Redox regulation of forkhead proteins through a p66shc-dependent signaling pathway. Science.

[160] Das F., Ghosh-Choudhury N., Dey N., Bera A., Mariappan M.M., Kasinath B.S., Ghosh Choudhury G. (2014). High Glucose Forces a Positive Feedback Loop Connecting Akt Kinase and FoxO1 Transcription Factor to Activate mTORC1 Kinase for Mesangial Cell Hypertrophy and Matrix Protein Expression. J. Biol. Chem..

[161] Huang W., Qiu J., Navarro I., Gonzalez P., Challa P. (2010). FOXO Protein Expression is Down-Regulated With Aging in the Lens. Investig. Ophthalmol. Vis. Sci..

[162] Rose-John S., Winthrop K., Calabrese L. (2017). The role of IL-6 in host defence against infections: Immunobiology and clinical implications. Nat. Rev. Rheumatol..

[163] Dethlefsen C., Højfeldt G., Hojman P. (2013). The role of intratumoral and systemic IL-6 in breast cancer. Breast Cancer Res. Treat..

[164] Gabay C. (2006). Interleukin-6 and chronic inflammation. Arthritis Res. Ther..

[165] Chen W., Lin H., Zhong X., Liu Z., Geng Y., Xie C., Chen W. (2014). Discrepant expression of cytokines in inflammation- and age-related cataract patients. PLoS ONE.

[166] Potvin S., Stip E., Sepehry A.A., Gendron A., Bah R., Kouassi E. (2008). Inflammatory cytokine alterations in schizophrenia: A systematic quantitative review. Biol. Psychiatry.

[167] Patterson P.H. (2009). Immune involvement in schizophrenia and autism: Etiology, pathology and animal models. Behav. Brain Res..

[168] Mohamed-Ali V., Goodrick S., Rawesh A., Katz D.R., Miles J.M., Yudkin J.S., Klein S., Coppack S.W. (1997). Subcutaneous adipose tissue releases interleukin-6, but not tumor necrosis factor-alpha, in vivo. J. Clin. Endocrinol. Metab..

[169] Straub R.H., Hense H.W., Andus T., Scholmerich J., Riegger G.A., Schunkert H. (2000). Hormone replacement therapy and interrelation between serum interleukin-6 and body mass index in postmenopausal women. J. Clin. Endocrinol. Metab..

[170] Fernandez-Real J.M., Vayreda M., Richart C., Gutierrez C., Broch M., Vendrell J., Ricart W.J. (2001). Circulating interleukin 6 levels, blood pressure, and insulin sensitivity in apparently healthy men and women. J. Clin. Endocrinol. Metab..

[171] Kado S., Nagase T., Nagata N. (1999). Circulating levels of interleukin-6, its soluble receptor and interleukin-6/interleukin-6 receptor complexes in patients with type 2 diabetes mellitus. Acta Diabetol..

[172] Pickup J.C., Mattock M.B., Chusney G.D., Burt D. (1997). NIDDM as a disease of the innate immune system: Association of acute-phase reactants and interleukin-6 with metabolic syndrome X. Diabetologia.

[173] Sumegová K., Nagyová Z., Waczulíková I., Žitňanová I., Ďuračková Z. (2007). Activity of Paraoxonase 1 and Lipid Profile in Healthy Children. Physiol. Res..

[174] Rozenberg O., Shiner M., Aviram M., Hayek T. (2008). Paraoxonase 1 (PON1) attenuates diabetes development in mice through its antioxidative properties. Free Rad. Biol. Med..

[175] Koren-Gluzer M., Aviram M., Meilin E., Hayek T. (2011). The antioxidant HDL-associated paraoxonase-1 (PON1) attenuates diabetes development and stimulates β-cell insulin release. Atherosclerosis.

[176] Baskol G., Karakucuk S., Oner A.O., Baskol M., Kocer D., Mirza E., Saraymen R., Ustdal M. (2006). Serum paraoxonase 1 activity and lipid peroxidation levels in patients with age-related macular degeneration. Ophthalmologica.

[177] Ates O., Azizi S., Alp H.H., Kiziltunc A., Beydemir S., Cinici E., Kocer I., Baykal O. (2009). Decreased serum paraoxonase 1 activity and increased serum homocysteine and malondialdehyde levels in age-related macular degeneration. Tohoku J. Exp. Med..

[178] Javadzadeh A., Ghorbanihaghjo A., Bahreini E., Rashtchizadeh N., Argani H., Alizadeh S. (2012). Serum paraoxonase phenotype distribution in exudative age-related macular degeneration and its relationship to homocysteine and oxidized low-density lipoprotein. Retina.

[179] Hashim Z., Zarina S. (2007). Assessment of paraoxonase activity and lipid peroxidation levels in diabetic and senile subjects suffering from cataract. Clin. Biochem..

[180] Butterfield D.A., Lange M.L. (2009). Multifunctional roles of enolase in Alzheimer’s disease brain: Beyond altered glucose metabolism. J. Neurochem..

[181] Warburg O., Christian W. (1942). Isolation and crystallization of enolase. Biochem. Z..

[182] Cimasoni G. (1972). The Inhibition of Enolase by Fluoride in vitro. Caries Res..

[183] Qin J., Chai G., Brewer J.M., Lovelace L.L., Lebioda L. (2006). Fluoride inhibition of enolase: Crystal structure and thermodynamics. Biochemistry.

[184] Pietkiewicz J., Gamian A., Staniszewska M., Danielewicz R. (2009). Inhibition of human muscle-specific enolase by methylglyoxal and irreversible formation of advanced glycation end products. J. Enzyme Inhib. Med. Chem..

[185] Chibber R., Molinatti P.A., Rosatto N., Lambourne B., Kohner E.M. (1997). Toxic action of advanced glycation end products on cultured retinal capillary pericytes and endothelial cells: Revelance to diabetic retinopathy. Diabetologia.

[186] Duhaiman A.S. (1995). Glycation of human lens proteins from diabetic and nondiabetic senile cataract patients. Glycoconj. J..

[187] Zhang K.L., Lou D.D., Guan Z.Z. (2015). Activation of the AGE/RAGE system in the brains of rats and in SH-SY5Y cells exposed to high level of fluoride might connect to oxidative stress. Neurotoxicol. Teratol..

[188] Wistow G.J., Lietman T., Williams L.A., Stapel S.O., De Jong W.W., Horwitz J., Piatigorsky J. (1988). τ-Crystallin/α-enolase: One gene encodes both an enzyme and a lens structural protein. J. Cell Biol..

[189] Iida H., Yahara I. (1985). Yeast heat-shock protein of Mr 48,000 is an isoprotein of enolase. Nature.

[190] Aaronson R.M., Graven K.K., Tucci M., McDonald R.J., Farber H.W. (1995). Non-neuronal enolase is an endothelial hypoxic stress protein. J. Biol. Chem..

[191] Subramanian A., Miller D.M. (2000). Structural analysis of α-enolase. Mapping the functional domains involved in down-regulation of the c-myc protooncogene. J. Biol. Chem..

[192] Piatigorsky J. (2003). Gene Sharing, Lens Crystallins and Speculations on an Eye/Ear Evolutionary Relationship. Integr. Comp. Biol..

[193] Zigler J.S., Sinha D. (2014). βA3/A1-crystallin: More than a lens protein. Prog. Retin. Eye Res..

[194] Urbak L., Vorum H. (2011). Heat shock proteins in the human eye. Int. J. Proteom..

[195] Bagchi M., Ireland M., Katar M., Maisel H. (2001). Heat shock proteins of chicken lens. J. Cell. Biochem..

[196] Strunnikova N., Baffi J., Gonzalez A., Silk W., Cousins S.W., Csaky K.G. (2001). Regulated heat shock protein 27 expression in human retinal pigment epithelium. Investig. Ophthalmol. Vis. Sci..

[197] Hoehenwarter W., Tang Y., Ackermann R., Pleissner K.P., Schmid M., Stein R., Zimny-Arndt U., Kumar N.M., Jungblut P.R. (2008). Identification of proteins that modify cataract of mouse eye lens. Proteomics.

[198] Salehi Z., Gholaminia Z., Panjtanpanah M.R. (2017). Association of HSP70-2 Gene 1267A/G Polymorphism with Cataract Incidence Among Guilan Population. Iran South Med. J..

[199] Zhang Y., Gong J., Zhang L., Xue D., Liu H., Liu P. (2013). Genetic polymorphisms of HSP70 in age-related cataract. Cell Stress Chaperones..

[200] Ayub H., Khan M.I., Micheal S., Akhtar F., Ajmal M., Shafique S., Benish Ali S.H., den Hollander A.I., Ahmed A., Qamar R. (2010). Association of eNOS and HSP70 gene polymorphisms with glaucoma in Pakistani cohorts. Mol. Vis..

[201] Harris L.L., Talian J.C., Zelenka P.S. (1992). Contrasting patterns of c-myc and N-myc expression in proliferating, quiescent, and differentiating cells of the embryonic chicken lens. Development.

[202] Cavalheiro G.R., Matos-Rodrigues G.E., Gomes A.L., Rodrigues P.M.G., Martins R.A.P. (2014). c-myc Regulates Cell Proliferation during Lens Development. PLoS ONE.

[203] Ashery-Padan R., Marquardt T., Zhou X., Gruss P. (2000). Pax6 activity in the lens primordium is required for lens formation and for correct placement of a single retina in the eye. Genes Dev..

[204] Panneerselvam L., Raghunath A., Perumal E. (2017). Differential expression of myocardial heat shock proteins in rats acutely exposed to fluoride. Cell Stress Chaperones..

[205] Zhao Y., Zhao J., Wang J., Wang J. (2017). Fluoride exposure changed the structure and the expressions of HSP related genes in testes of pubertal rats. Chemosphere.

[206] Chattopadhyay A., Podder S., Agarwal S., Bhattacharya S. (2011). Fluoride-induced histopathology and synthesis of stress protein in liver and kidney of mice. Arch. Toxicol..

[207] Chen Q., Wang Z., Xiong Y., Xue W., Kao X., Gao Y., Muhammad N., Song D. (2009). Selenium increases expression of HSP70 and antioxidant enzymes to lessen oxidative damage in Fincoal-type fluorosis. J. Toxicol. Sci..

[208] Waugh D.T. (2019). Molecular Mechanisms of Fluoride inhibition of Na+, K+-ATPase activity: Implications for Public Health and Health Inequalities. Int. J. Environ. Res. Public Health.

[209] Opit L.J., Potter H., Charnock J.S. (1966). The effect of anions on (Na+ + K+)-activated. ATPase. Biochim. Biophys. Acta.

[210] Yoshida H., Nagai K., Kamei M., Nakagawa Y. (1968). Irreversible inactivation of (Na+-K+)-dependent ATPase and K+-dependent phosphatase by fluoride. Biochim. Biophys. Acta.

[211] Millman M.S., Omachi A. (1972). The Role of Oxidized Nicotinamide Adenine Dinucleotide in Fluoride Inhibition of Active Sodium Transport in Human Erythrocytes. J. Gen. Physiol..

[212] Robinson J.D., Davis R.L., Steinberg M. (1986). Fluoride and beryllium interact with the (Na + Kbdependent ATPase as analogs of phosphate. J. Bioenergy Biomembr..

[213] Murphy A.J., Hoover J.C. (1992). Inhibition of the Na/K-ATPase by fluoride. Parallels with its inhibition of the sarcoplasmic reticulum CaATPase. J. Biol. Chem..

[214] Façanha A.R., de Meis L. (1995). Inhibition of Maize Root H+-ATPase by Fluoride and Fluoroaluminate Complexes. Plant Physiol..

[215] Swann A.C. (1990). Inhibition of (Na+,K+)-ATPase by fluoride: Evidence for a membrane adaptation to ethanol. Alcohol.

[216] Suketa Y., Suzuki K., Taki T., Itoh Y., Yamaguchi M., Sakurai T., Tanishita Y. (1995). Effect of fluoride on the activities of the Na+/glucose cotransporter and Na+/K(+)-ATPase in brush border and basolateral membranes of rat kidney (in vitro and in vivo). Biol. Pharm. Bull..

[217] Iukhnovets R.A., Bachinskiĭ P.P. (1982). Effect of fluoride and insulin on cation-dependent ATPase activity of the enterocytes during threonine absorption. Vopr. Med. Khim..

[218] Zhan X.A., Li J.X., Wang M., Xu Z.R. (2006). Effects of Fluoride on Growth and Thyroid Function in Young Pigs. Fluoride.

[219] Sarkar C., Pal S. (2014). Ameliorative effect of resveratrol against fluoride-induced alteration of thyroid function in male wistar rats. Biol. Trace Elem. Res..

[220] Sarkar C., Pal S. (2015). Effects of sub-acute fluoride exposure on discrete regions of rat brain associated with thyroid dysfunction: A comparative study. Int. J. Biomed. Res..

[221] Arulkumar M., Vijayan R., Penislusshiyan S., Sathishkumar P., Angayarkanni J., Palvannan T. (2017). Alteration of paraoxonase, arylesterase and lactonase activities in people around fluoride endemic area of Tamil Nadu, India. Clin. Chim. Acta.

[222] Shashi A., Meenakshi G. (2015). Inhibitory Effect of Fluoride on Na+,K+ ATPase Activity in Human Erythrocyte Membrane. Biol. Trace Elem. Res..

[223] Gallicchio M.A., Bach L.A. (2010). Advanced glycation end products inhibit Na+ K+ ATPase in proximal tubule epithelial cells: Role of cytosolic phospholipase A2alpha and phosphatidylinositol 4-phosphate 5-kinase gamma. Biochim. Biophys. Acta.

[224] Thangapandiyan S., Miltonprabu S. (2015). Epigallocatechin gallate exacerbates fluoride-induced oxidative stress mediated testicular toxicity in rats through the activation of Nrf2 signaling pathway. Asian Pacif. J. Reprod..

[225] Janowiak B.E., Hayward M.A., Peterson F.C., Volkman B.F., Griffith O.W. (2006). Gamma-glutamylcysteine synthetase-glutathione synthetase: Domain structure and identification of residues important in substrate and glutathione binding. Biochemistry.

[226] Carnicer M.J., Bernardini S., Bellincampi L., Noguera N.I., Nuccetelli M., Ammatuna E., Breccia M., Lo-Coco F., Federici G. (2006). Role of gamma-glutamyl cysteine synthetase (gamma-GCS) gene expression as marker of drug sensitivity in acute myeloid leukemias. Clin. Chim. Acta.

[227] Patil K., Bellner L., Cullaro G., Gotlinger K.H., Dunn M.W., Schwartzman M.L. (2008). Heme oxygenase-1 induction attenuates corneal inflammation and accelerates wound healing after epithelial injury. Investig. Ophthalmol. Vis. Sci..

[228] Nath D.A., Balla G., Vercelotti G.M., Balla J., Jacob H.S., Levitt M.D., Rosenberg M.E. (1992). Induction of heme oxygenase is a rapid, protective response in rhabdomyolysis in the rat. J. Clin. Investig..

[229] Choi A.M.K., Alam J. (1996). Heme Oxygenase-1: Function, Regulation, and Implication of a Novel Stress-inducible Protein in Oxidant-induced Lung Injury. Am. J. Respir. Cell. Mol. Biol..

[230] Seta F., Bellner L., Rezzani R., Regan R.F., Dunn M.W., Abraham N.G., Gronert K., Laniado-Schwartzman M. (2006). Heme oxygenase-2 is a critical determinant for execution of an acute inflammatory and reparative response. Am. J. Pathol..

[231] Tiwari S., Gupta S.K., Kumar K., Trivedi R., Godbole M.M. (2004). Simultaneous exposure of excess fluoride and calcium deficiency alters VDR, CaR, and calbindin D 9 k mRNA levels in rat duodenal mucosa. Calcif. Tissue Int..

[232] Sun J., Kong J., Duan Y., Szeto F.L., Liao A., Madara J.L., Li Y.C. (2006). Increased NF-kappaB activity in fibroblasts lacking the vitamin D receptor. Am. J. Physiol. Endocrinol. Metab..

[233] Stachowska E., Baśkiewicz-Masiuk M., Machaliński B., Rybickam M., Gutowska I., Bober J., Grymula K., Dziedziejko V., Chlubek D. (2005). Sodium fluoride enhancement of monocyte differentiation via nuclear factor κB mechanism. Fluoride.

[234] Chen Q., Wang Z., Xiong Y., Zou X., Liu Z. (2010). Comparative study of p38 MAPK signal transduction pathway of peripheral blood mononuclear cells from patients with coal-combustion-type fluorosis with and without high hair selenium levels. Int. J. Hyg. Environ. Health.

[235] Tian Y., Huo M., Li G., Wang J. (2016). Regulation of LPS-induced mRNA expression of pro-inflammatory cytokines via alteration of NF-κB activity in mouse peritoneal macrophages exposed to fluoride. Chemosphere.

[236] Jones E., Adcock I.M., Ahmed B.Y., Punchard N.A. (2007). Modulation of LPS stimulated NF-kappaB mediated Nitric Oxide production by PKCε and JAK2 in RAW macrophages. J. Inflamm. (Lond.).

[237] Luo Q., Cui H., Deng H., Kuang P., Liu H., Lu Y., Fang J., Zuo Z., Deng J., Li Y. (2017). Sodium fluoride induces renal inflammatory responses by activating NF-κB signaling pathway and reducing anti-inflammatory cytokine expression in mice. Oncotarget.

[238] Refsnes M., Skuland T., Låg M., Schwarze P.E., Øvrevik J. (2014). Differential NF-κB and MAPK activation underlies fluoride- and TPA-mediated CXCL8 (IL-8) induction in lung epithelial cells. J. Inflamm. Res..

[239] Deng H., Kuang P., Cui H., Lou Q., Liu H., Lu Y., Fang J., Zuo Z., Deng J., Li Y. (2017). Sodium fluoride induces apoptosis in mouse splenocytes by activating ROS-dependent NF-κB signalling. Oncotarget.

[240] Zhang M., Wang A., Xia T., He P. (2008). Effects of fluoride on DNA damage, S-phase cell-cycle arrest and the expression of NF-kappaB in primary cultured rat hippocampal neurons. Toxicol. Lett..

[241] Zhang J., Zhu W.J., Xu X.H., Zhang Z.G. (2011). Effect of fluoride on calcium ion concentration and expression of nuclear transcription factor kappa-B ρ65 in rat hippocampus. Exp. Toxicol. Pathol..

[242] Misra U.K., Gawdi G., Pizzo S.V. (2002). Beryllium fluoride-induced cell proliferation: A process requiring P21(ras)-dependent activated signal transduction and NF-kappaB-dependent gene regulation. J. Leukoc. Biol..

[243] Shanmugam T., Abdulla S., Yakulasamy V., Selvaraj M., Mathan R. (2018). A mechanism underlying the neurotoxicity induced by sodium fluoride and its reversal by epigallocatechin gallate in the rat hippocampus: Involvement of NrF2/Keap-1 signaling pathway. J. Basic Appl. Zool..

[244] Yan N., Liu Y., Liu S. (2016). Fluoride-Induced Neuron Apoptosis and Expressions of Inflammatory Factors by Activating Microglia in Rat Brain. Mol. Neurobiol..

[245] Sun Y., Ke L., Zheng X., Li T., Ouyang W., Zhang Z. (2017). Effects of Different Levels of Calcium Intake on Brain Cell Apoptosis in Fluorosis Rat Offspring and Its Molecular Mechanism. Biol. Trace Elem. Res..

[246] Zhang J., Zhang Z. (2013). Effects of chronic fluorosis on CAMKIIα, C-FOS, BAX, and BCL-2 channel signalling in the Hippocampus of Rats. Fluoride.

[247] Zhang W.L., Cui Y.N., Gao S., Zhang X.Y., Li G.S. (2003). Expression of proto-oncogenes c-fos and c-jun in osteoblasts activated by excessive fluoride. Zhonghua Yu Fang Yi Xue Za Zhi.

[248] Teng Y., Zhang J., Zhang Z., Feng J. (2018). The Effect of Chronic Fluorosis on Calcium Ions and CaMKIIα, and c-fos Expression in the Rat Hippocampus. Biol. Trace Elem. Res..

[249] Poche R.A., Sharma R., Garcia M.D., Wada A.M., Nolte M.J., Udan R.S., Paik J.H., DePinho R.A., Bartlett J.D., Dickinson M.E. (2012). Transcription factor FoxO1 is essential for enamel biomineralization. PLoS ONE.

[250] Gao J., Ruan J., Gao L. (2014). Excessive fluoride reduces Foxo1 expression in dental epithelial cells of the rat incisor. Eur. J. Oral Sci..

[251] Li J., Zhao L., Zhao X., Wang P., Liu Y., Ruan J. (2017). Foxo1 attenuates NaF-induced apoptosis of LS8 cells through the JNK and mitochondrial pathways. Biol. Trace Elem. Res..

[252] Li J., Wang P., Gao J., Fei X., Liu Y., Ruan J. (2018). NaF Reduces KLK4 Gene Expression by Decreasing Foxo1 in LS8 Cells. Biol. Trace Elem. Res..

[253] Liu J., Xia T., Zhang M., He W., He P., Chen X., Yang K., Wang A. (2006). Screening of Environmental Response Genes Related To Dental Fluorosis. Fluoride.

[254] Akashi M., Loussararian A.H., Adelman D.C., Saito M., Koeffler H.P. (1990). Role of lymphotoxin in expression of interleukin 6 in human fibroblasts. Stimulation and regulation. J. Clin. Investig..

[255] Refsnes M., Becher R., Lag M., Skuland T., Schwarze P.E. (1999). Fluoride-induced interleukin-6 and interleukin-8 synthesis in human epithelial lung cells. Hum. Exp. Toxicol..

[256] Afolabi O.K., Oyewo E.B., Adekunle A.S., Adedosu O.T., Adedeji A.L. (2013). Oxidative indices correlate with dyslipidemia and pro-inflammatory cytokine levels in fluoride-exposed rats. Arh. Hig. Rada Toksikol..

[257] Ma Y., Niu R., Sun Z., Wang J., Luo G., Zhang J., Wang J. (2012). Inflammatory responses induced by fluoride and arsenic at toxic concentration in rabbit aorta. Arch. Toxicol..

[258] Schwarze P.E., Låg M., Becher R., Thrane E.V., Samuelsen J.T., Hetland R.B., Refsnes M. (2000). Role of signal transduction pathways in lung inflammatory responses. Toxicol. Lett..

[259] Chouhan S., Lomash V., Flora S.J.S. (2010). Fluoride-induced changes in haem biosynthesis pathway, neurological variables and tissue histopathology of rats. J. Appl. Toxicol..

[260] Kleiner H.S., Allmann D.W. (1982). The effects of fluoridated water on rat urine and tissue cAMP levels. Arch. Oral Biol..

[261] Raina R., Baba N.A., Verma P.K., Sultana M., Singh M. (2015). Hepatotoxicity Induced by Subchronic Exposure of Fluoride and Chlorpyrifos in Wistar Rats: Mitigating Effect of Ascorbic Acid. Biol. Trace Elem. Res..

[262] Baba N.A., Raina R., Verma P.K., Sultana M., Prawez S., Nisara N.A. (2013). Toxic effects of fluoride and chlorpyrifos on antioxidant parameters in rats: Protective effects of vitamins C and E. Fluoride.

[263] Chlubek D., Grucka-Mamczar E., Birkner E., Polaniak R., Starwiarska-Pieta B., Duliban H. (2003). Activity of pancreatic antioxidative enzymes and malondialdehyde concentrations in rats with hyperglycemia caused by fluoride intoxication. J. Trace Elem. Med. Biol..

[264] Viglino P., Rigo A., Stevanato R., Ranieri G.A. (1969). The binding of fluoride ion to bovine cuprozinc superoxide dismutase as studied by 19F magnetic relaxation. J. Magn. Resonance.

[265] Dooley D.M., Jones T.F., Karas J.L., McGuirl M.A., Brown R.D., Koenig S.H. (1987). Azide and fluoride binding to E. coli iron superoxide dismutase as studied by solvent proton magnetic relaxation dispersion. J. Am. Chem. Soc..

[266] Varol E., Icli A., Aksoy F., Bas H.A., Sutcu R., Ersoy I.H., Varol S., Ozaydin M. (2013). Evaluation of total oxidative status and total antioxidant capacity in patients with endemic fluorosis. Toxicol. Ind. Health.

[267] Kalyanalakshmi P., Vijayabhaskar M., Dhananjaya Naidu M. (2007). Lipid peroxidation and antioxidant enzyme status of adult males with skeletal fluorosis in Andhra Pradesh, India. Fluoride.

[268] Shanthakumari D., Srinivasalu S., Subramanian S. (2006). Antioxidant defense systems in red blood cell lysates of men with dental fluorosis living in Tamil Nadu, India. Fluoride.

[269] Kumari D.S., Rao P.R. (1991). Red cell membrane alterations in human chronic fluoride toxicity. Biochem. Int..

[270] Shivarajashankara Y.M., Shivashankara A.R., Gopalakrishna B.P., Rao S.H. (2011). Oxidative stress in children with endemic skeletal fluorosis. Fluoride.

[271] Reddy G.B., Khandare A.L., Reddy P.Y., Rao G.S., Balakrishna N., Srivalli I. (2003). Antioxidant defense system and lipid peroxidation in patients with skeletal fluorosis and in fluoride-intoxicated rabbits. Toxicol. Sci..

[272] Gavriliuc L., Stepco E., Lupan I., Sevcenco N., Spine I. (2012). Salivary Glutathione-Dependent Enzymes in Patients with Dental Fluorosis Treated by Complex Antioxidant Therapy. Balk J. Stom.

[273] Anwar A.J., Walker J.D., Frier B.M. (1998). Type 1 diabetes mellitus and Down’s syndrome: Prevalence, management and diabetic complications. Diabet. Med..

[274] Bergholdt R., Eising S., Nerup J., Pociot F. (2006). Increased prevalence of Down’s syndrome in individuals with type 1 diabetes in Denmark: A nationwide population-based study. Diabetologia.

[275] Prasher V.P. (1995). Prevalence of psychiatric disorders in adults with Down’s syndrome. Eur. J. Psychiatry.

[276] Ekstein S., Glick B., Weill M., Kay B., Berger I. (2011). Down’s syndrome and attention-deficit/hyperactivity disorder (ADHD). J. Child Neurol..

[277] Vicari S., Pontillo M., Armando M. (2013). Neurodevelopmental and psychiatric issues in Down’s syndrome: Assessment and intervention. Psychiatr. Genet..

[278] Dykens E.M., Shah B., Davis B., Baker C., Fife T., Fitzpatrick J. (2015). Psychiatric disorders in adolescents and young adults with Down’s syndrome and other intellectual disabilities. J. Neurodev. Disord..

[279] Tassé M.J., Navas Macho P., Havercamp S.M., Benson B.A., Allain D.C., Manickam K., Davis S. (2016). Psychiatric Conditions Prevalent Among Adults with Down’s syndrome. J. Pol. Pract. Intellect. Disabil..

[280] Oxelgren U.W., Myrelid Å., Annerén G., Ekstam B., Göransson C., Holmbom A., Isaksson A., Åberg M., Gustafsson J., Fernell E. (2017). Prevalence of autism and attention-deficit-hyperactivity disorder in Down’s syndrome: A population-based study. Dev. Med. Child Neurol..

[281] Kohen D. (2004). Diabetes mellitus and schizophrenia: Historical perspective. Br. J. Psychiatry Suppl..

[282] Dixon L., Weiden P., Delahanty J., Goldberg R., Postrado L., Lucksted A., Lehman A. (2000). Prevalence and correlates of diabetes in national schizophrenia samples. Schizophr. Bull..

[283] De Hert M., van Winkel R., Van Eyck D., Hanssens L., Wampers M., Scheen A., Peuskens J. (2006). Prevalence of diabetes, metabolic syndrome and metabolic abnormalities in schizophrenia over the course of the illness: A cross-sectional study. Clin. Pract. Epidemol. Ment. Health.

[284] Fatemi S.H., Stary J.M., Halt A.R., Realmuto G.R. (2001). Dysregulation of Reelin and Bcl-2 proteins in autistic cerebellum. J. Autism. Dev. Disord..

[285] Fatemi S.H., Halt A.R., Stary J.M., Realmuto G.M., Jalali-Mousavi M. (2001). Reduction in anti-apoptotic protein Bcl-2 in autistic cerebellum. Neuroreport.

[286] Araghi-Niknam M., Fatemi S.H. (2003). Levels of Bcl-2 and P53 are altered in superior frontal and cerebellar cortices of autistic subjects. Cell. Mol. Neurobiol..

[287] Mal’tseva V.A. (1973). Eye damage in workers with fluorine intoxication. Vestn. Oftalmol..

[288] Karczewicz D., Baranowska-George T., Palacz O., Tokarz-Sawinska E., Stankiewicz W., Krzystolik Z., Lubinski W., Kosmider K. (1989). Evaluation of the visual system in peoplehaving extended contact with fluorine. Klin. Oczna.

[289] Liu I.Y., White L., LaCroix A.Z. (1989). The association of age-related macular degeneration and lens opacities in the aged. Am. J. Public Health.

[290] Lin C.C., Lin C.Y., Ma H.Y. (2000). Pulmonary function changes and increased Th-2 cytokine expression and nuclear factor kB activation in the lung after sensitization and allergen challenge in brown Norway rats. Immunol. Lett..

[291] Caramori G., Casolari P., Adcock I. (2013). Role of Transcription Factors in the Pathogenesis of Asthma and COPD. Cell Commun. Adhes..

[292] Caramori G., Adcock I.M., Ito K. (2004). Anti-inflammatory inhibitors of IkappaB kinase in asthma and COPD. Curr. Opin. Investig. Drugs.

[293] Edwards M.R., Bartlett N.W., Clarke D., Birrell M., Belvisi M., Johnston S.L. (2009). Targeting the NF-kappaB pathway in asthma and chronic obstructive pulmonary disease. Pharmacol. Ther..

[294] Kim M.K., Chung S.W., Kim D.H., Kim J.M., Ha Y.M., Kim Y.H., No J.K., Chung H.S., Park K.Y., Rhee S.H. (2010). Modulation of age-related NF-kappaB activation by dietary zingerone via MAPK pathway. Exp. Gerontol..

[295] Naik U.S., Gangadharan C., Abbagani K., Nagalla B., Dasari N., Manna S.K. (2011). A study of nuclear transcription factor-kappa B in childhood autism. PLoS ONE.

[296] Young A.M., Campbell E., Lynch S., Suckling J., Powis S.J. (2011). Aberrant NF-kappaB expression in autism spectrum condition: A mechanism for neuroinflammation. Front. Psychiatry..

[297] Abdel-Salam O.M.E., Youness E.R., Mohammed N.A., Elhamed W.A.A. (2015). Nuclear Factor-Kappa B and Other Oxidative Stress Biomarkers in Serum of Autistic Children. Open J. Mol. Integr. Physiol..

[298] Ghanizadeh A. (2011). Nuclear factor kappa B may increase insight into the management of neuroinflammation and excitotoxicity in autism. Expert Opin. Ther. Targets.

[299] Feng Y., Li X., Zhou W., Lou D., Huang D., Li Y., Kang Y., Xiang Y., Li T. (2016). Regulation of set gene expression by NF- κB. Mol. Neurobiol..

[300] Lukiw W.J., Bazan N.G. (1998). Strong nuclear factor-κB-DNA binding parallels cyclooxygenase-2 gene transcription in aging and in sporadic Alzheimer’s disease superior temporal lobe neocortex. J. Neurosci. Res..

[301] Boissiere F., Hunot S., Faucheux B., Duyckaerts C., Hauw J.J., Agid Y., Hirsch E.C. (1997). Nuclear translocation of NF-κB in cholinergic neurons of patients with Alzheimer’s disease. Neuroreport.

[302] Akiyama H., Nishimura T., Kondo H., Ikeda K., Hayashi Y., McGeer P.L. (1994). Expression of the receptor for macrophage colony stimulating factor by brain microglia and its upregulation in brains of patients with Alzheimer’s disease and amyotrophic lateral sclerosis. Brain Res..

[303] Hunot S., Brugg B., Ricard D., Michel P.P., Muriel M.P., Ruberg M., Faucheux B.A., Agid Y., Hirsch E.C. (1997). Nuclear translocation of NF-κB is increased in dopaminergic neurons of patients with Parkinson disease. Proc. Natl. Acad. Sci. USA.

[304] Block M.L., Hong J.S. (2005). Microglia and inflammation-mediated neurodegeneration: Multiple triggers with a common mechanism. Prog. Neurobiol..

[305] Bonetti B., Stegagno C., Cannella B., Rizzuto N., Moretto G., Raine C.S. (1999). Activation of NF-κB and c-jun transcription factors in multiple sclerosis lesions. Implications for oligodendrocyte pathology. Am. J. Pathol..

[306] Glass C.K., Saijo K., Winner B., Marchetto M.C., Gage F.H. (2010). Mechanisms underlying inflammation in neurodegeneration. Cell.

[307] Kim J.J., Mandelli L., Lim S., Lim H.K., Kwon O.J., Pae C.U., Serretti A., Nimgaonkar V.L., Paik I.H., Jun T.Y. (2008). Association analysis of heat shock protein 70 gene polymorphisms in schizophrenia. Eur. Arch. Psychiatry Clin. Neurosci..

[308] Kowalczyk M., Owczarek A., Suchanek R., Paul-Samojedny M., Fila-Danilow A., Borkowska P., Kucia K., Kowalski J. (2014). Heat shock protein 70 gene polymorphisms are associated with paranoid schizophrenia in the Polish population. Cell Stress Chaperones.

[309] El-Ansary A., Al-Ayadhi L. (2012). Neuroinflammation in autism spectrum disorders. J. Neuroinflamm..

[310] Aron Y., Busson M., Polla B.S., Dusser D., Lockhart A., Swierczewski E., Favatier F. (1999). Analysis of hsp70 gene polymorphism in allergic asthma. Allergy.

[311] Hou C., Zhao H., Li W., Zhenyu L., Dan Z., Laiyu L., Wancheng T., Shao-xi C., Fei Z. (2011). Increased heat shock protein 70 levels in induced sputum and plasma correlate with severity of asthma patients. Cell Stress Chaperones.

[312] Schett G., Redlich K., Xu Q., Bizan P., Groger M., Tohidast-Akrad M., Kiener H., Smolen J., Steiner G. (1998). Enhanced expression of heat shock protein 70 (hsp70) and heat shock factor 1 (HSF1) activation in rheumatoid arthritis synovial tissue. Differential regulation of hsp70 expression and hsf1 activation in synovial fibroblasts by proinflammatory cytokines, shear stress, and antiinflammatory drugs. J. Clin. Investig..

[313] Luo X., Zuo X., Zhang B., Song L., Wei X., Zhou Y., Xiao X. (2008). Release of heat shock protein 70 and the effects of extracellular heat shock protein 70 on the production of IL-10 in fibroblast-like synoviocytes. Cell Stress Chaperones.

[314] Najafizadeh S.R., Ghazizadeh Z., Nargesi A.A., Mahdavi M., Abtahi S., Mirmiranpour H., Nakhjavani M. (2015). Analysis of serum heat shock protein 70 (HSPA1A) concentrations for diagnosis and disease activity monitoring in patients with rheumatoid arthritis. Cell Stress Chaperones.

[315] Kang E.H., Kim D.J., Lee E.Y., Lee Y.J., Lee E.B., Song Y.W. (2009). Downregulation of heat shock protein 70 protects rheumatoid arthritis fibroblast-like synoviocytes from nitric oxide-induced apoptosis. Arthritis Res. Ther..

[316] Ucisik-Akkaya E., Davis C.F., Gorodezky C., Alaez C., Dorak M.T. (2010). HLA complex-linked heat shock protein genes and childhood acute lymphoblastic leukemia susceptibility. Cell Stress Chaperones.

[317] Ciocca D.R., Clark G.M., Tandon A.K., Fuqua S.A., Welch W.J., McGuire W.L. (1993). Heat shock protein hsp70 in patients with axillary lymph node-negative breast cancer: Prognostic implications. J. Natl. Cancer Inst..

[318] Hwang T.S., Han H.S., Choi H.K., Lee Y.J., Kim Y.J., Han M.Y., Park Y.M. (2003). Differential, stage-dependent expression of Hsp70, Hsp110 and Bcl-2 in colorectal cancer. J. Gastroenterol. Hepatol..

[319] Joo M., Chi J.G., Lee H. (2005). Expressions of HSP70 and HSP27 in hepatocellular carcinoma. J. Korean Med. Sci..

[320] Luk J.M., Lam C.T., Siu A.F., Lam B.Y., Ng I.O., Hu M.Y., Che C.M., Fan S.T. (2006). Proteomic profiling of hepatocellular carcinoma in Chinese cohort reveals heat-shock proteins (Hsp27, Hsp70, GRP78) up-regulation and their associated prognostic values. Proteomics.

[321] Alaiya A.A., Oppermann M., Langridge J., Roblick U., Egevad L., Brindstedt S., Hellström M., Linder S., Bergman T., Jörnvall H. (2001). Identification of proteins in human prostate tumor material by two-dimensional gel electrophoresis and mass spectrometry. Cell Mol. Life Sci..

[322] Tang D., Khaleque M.A., Jones E.L., Theriault J.R., Li C., Wong W.H., Stevenson M.A., Calderwood S.K. (2005). Expression of heat shock proteins and heat shock protein messenger ribonucleic acid in human prostate carcinoma in vitro and in tumors in vivo. Cell Stress Chaperones.

[323] Wang X.P., Wang Q.X., Li H.Y., Chen R.F. (2005). Heat shock protein 70 chaperoned alpha-fetoprotein in human hepatocellular carcinoma cell line BEL-7402. World J. Gastroenterol..

[324] Hellman K., Alaiya A.A., Schedvins K., Steinberg W., Hellstrom A.C., Auer G. (2004). Protein expression patterns in primary carcinoma of the vagina. Br. J. Cancer.

[325] Meng L., Hunt C., Yaglom J.A., Gabai V.L., Sherman M.Y. (2011). Heat shock protein Hsp72 plays an essential role in Her2-induced mammary tumorigenesis. Oncogene.

[326] Tetu B., Brisson J., Landry J., Huot J. (1995). Prognostic significance of heat-shock protein-27 in node-positive breast carcinoma: An immunohistochemical study. Breast Cancer Res. Treat..

[327] Kang S.H., Kang K.W., Kim K.H., Kwon B., Kim S.K., Lee H.Y., Kong S.Y., Lee E.S., Jang S.G., Yoo B.C. (2008). Upregulated HSP27 in human breast cancer cells reduces Herceptin susceptibility by increasing Her2 protein stability. BMC Cancer.

[328] Geisler J.P., Tammela J.E.J.E., Manahan K.J., Geisler H.E., Miller G.A.G.A., Zhou Z., Wiemann M.C. (2004). HSP27 in patients with ovarian carcinoma: Still an independent prognostic indicator at 60 months follow-up. Eur. J. Gynaecol. Oncol..

[329] Giaginis C., Daskalopoulou S.S., Vgenopoulou S., Sfiniadakis I., Kouraklis G., Theocharis S.E. (2009). Heat shock protein-27, -60 and -90 expression in gastric cancer: Association with clinicopathological variables and patient survival. BMC Gastroenterol..

[330] Foster C.S., Dodson A.R.A.R., Ambroisine L., Fisher G., Moller H., Clark J.J., Attard G., De-Bono J., Scardino P., Reuter V.E.V.E. (2009). Hsp-27 expression at diagnosis predicts poor clinical outcome in prostate cancer independent of ETS-gene rearrangement. Br. J. Cancer.

[331] Yu Z., Zhi J., Peng X., Zhong X., Xu A. (2010). Clinical significance of HSP27 expression in colorectal cancer. Mol. Med. Rep..

[332] Tweedle E.M., Khattak I., Ang C.W., Nedjadi T., Jenkins R., Park B.K., Kalirai H., Dodson A., Azadeh B., Terlizzo M. (2010). Low molecular weight heat shock protein HSP27 is a prognostic indicator in rectal cancer but not colon cancer. Gut.

[333] Ioachin E. (2005). Immunohistochemical tumour markers in endometrial carcinoma. Eur. J. Gynaecol. Oncol..

[334] Storm F.K., Mahvi D.M., Gilchrist K.W. (1993). Hsp-27 has no diagnostic or prognostic significance in prostate or bladder cancers. Urology.

[335] Ciocca D.R., Calderwood S.K. (2005). Heat shock proteins in cancer: Diagnostic, prognostic, predictive, and treatment implications. Cell Stress Chaperones..

[336] Tavernier E., Duval A., Cornillon J., Flandrin P., Guyotat D., Campos L. (2007). Prognostic value of CXCRCR4, adhesion molecules and heat shock proteins (HSP) in acute myelogenous leukemia. Blood (ASH Ann. Meet. Abstr.).

[337] Alexiou G.A., Karamoutsios A., Lallas G., Ragos V., Goussia A., Kyritsis A.P., Voulgaris S., Vartholomatos G. (2014). Expression of heat shock proteins in brain tumors. Turk. Neurosurg..

